# New cosurface capacitive stimulators for the development of active osseointegrative implantable devices

**DOI:** 10.1038/srep30231

**Published:** 2016-07-26

**Authors:** Marco P. Soares dos Santos, Ana Marote, T. Santos, João Torrão, A. Ramos, José A. O. Simões, Odete A. B. da Cruz e Silva, Edward P. Furlani, Sandra I. Vieira, Jorge A. F. Ferreira

**Affiliations:** 1 Centre for Mechanical Technology & Automation (TEMA), University of Aveiro, Aveiro, Portugal; 2 Department of Mechanical Engineering, University of Aveiro, Aveiro, Portugal; 3 Institute of Biomedicine (iBiMED), Department of Medical Sciences, University of Aveiro, Aveiro, Portugal; 4 Physics Department, University of Aveiro, Aveiro, Portugal; 5 Department of Chemical and Biological Engineering, University at Buffalo, SUNY, Buffalo, NY, US; 6 Department of Electrical Engineering, University at Buffalo, SUNY, Buffalo, NY, US

## Abstract

Non-drug strategies based on biophysical stimulation have been emphasized for the treatment and prevention of musculoskeletal conditions. However, to date, an effective stimulation system for intracorporeal therapies has not been proposed. This is particularly true for active intramedullary implants that aim to optimize osseointegration. The increasing demand for these implants, particularly for hip and knee replacements, has driven the design of innovative stimulation systems that are effective in bone-implant integration. In this paper, a new cosurface-based capacitive system concept is proposed for the design of implantable devices that deliver controllable and personalized electric field stimuli to target tissues. A prototype architecture of this system was constructed for *in vitro* tests, and its ability to deliver controllable stimuli was numerically analyzed. Successful results were obtained for osteoblastic proliferation and differentiation in the *in vitro* tests. This work provides, for the first time, a design of a stimulation system that can be embedded in active implantable devices for controllable bone-implant integration and regeneration. The proposed cosurface design holds potential for the implementation of novel and innovative personalized stimulatory therapies based on the delivery of electric fields to bone cells.

Musculoskeletal conditions are the second most common cause of global disability, affecting more than 20% of the world population^1^,^2^. Increasing incidences of musculoskeletal disorders have been observed in developed and emerging countries over the last 20 years^2^. This includes osteoarthritis, a major health problem with a global prevalence of approximately 4%, and the most common indication for both total hip and knee replacements^2^,^3^,^4^. Although both of these procedures are already among the most prevalent surgeries performed worldwide, upsurges in their incidences are predicted in the forthcoming decades^3^,^4^,^5^,^6^. The sustained increases in the incidences of knee and hip replacements are due mainly to ageing, sedentary lifestyle and obesity. These surgeries represent a burden for individuals, and health and social care systems^3^,^4^,^6^. Moreover, surgical revisions of hip and knee replacements are usually more complex and more invasive than primary replacements, and their revision rates may exceed 10%^7^,^8^,^9^. Hence, implants must be projected to minimize disability and avoid failures throughout the patients’ lifetime, which may represent implant lifetimes in excess of 50 years. The requirement of implants with improved durability is also emphasized by the higher deterioration risks of mid and long-term fixed cemented implants and by a significant increase in the number of more active and younger patients^10^.

Asymptomatic long-term fixation of orthopaedic implants requires their osseointegration^10^,^11^. Interlocking between bone and implant surfaces should be accomplished at the micrometer and nanometer scale levels^11^,^12^. However, bone loss is a frequent phenomenon that renders unstable bone-implant fixations^10^,^13^. This is significantly promoted by adverse bone remodeling in response to wear debris and stress-shielding and can result in aseptic loosening^10^,^13^. To avoid such implant failure^3^,^4^, effective regeneration of the peri-prosthetic bone stock is required to ensure a correct biologic interplay at nano- to macro-scale levels. The bone-implant topography and the presence of osseointegrative stimuli have a significant influence over this outcome, given their known influence on the biointegration mechanisms^11^,^13^. For optimum osseointegration to be achieved, these positive osseointegrative cues should be optimized.

Pre-optimization of the implants’ design is a current methodology used to improve their performance. Micro and nanometer-scale texture design of the implants’ surfaces, and custom-made geometries, are sophisticated methods that are used to maximize the host-implant responses^12^,^14^. Advanced biomaterials, inert and nearly-inert, have been sintered to improve the mechanical properties of the bone-implant interface, including bioceramics and biocompatible metals and alloys^15^. These approaches are nevertheless used to optimize passive implants, i.e., implants without resources to promote bioactivity. However, regardless of their materials, design, rehabilitation protocols, or the surgical procedures used, these passive implants were recently demonstrated to be unable to minimize failures throughout the implants’ lifetime^16^. For optimal performance to be achieved, novel implants should be able to perform trajectories from failure states to without-failure states^16^. Consequently, they must be biologically active throughout their lifetime^16^. Actuation systems based on biomaterials and drug-delivery systems are being explored to produce clinically-useful active implant systems. Innovative bioactive biomaterials for coating uncemented implants have been exhaustively researched^17^,^18^,^19^. Another promising approach to enhance osseointegration and prevent peri-operative infections is to impregnate coatings with drugs (gentamicin, tobramycin and vancomycin, etc.) and/or biomolecules (growth factors, collagen and proteins, etc.)^17^,^20^,^21^,^22^. These solutions can however present significant drawbacks for bone-implant optimal bonding, namely: (i) extremely complex designs may be required, mainly for multifunctional coatings^17^; (ii) their controllability is reduced, as their behavior cannot be changed after implant insertion; (iii) their delivery dynamics do not consider current biochemical and biomechanical states of the target bone tissues; (iv) their ability to perform personalized delivery is quite limited; (v) long-term release of bioactive substances by these implants is currently unfeasible and will most likely be quite difficult to implement in the forthcoming years; (vi) the simultaneous delivery of different stimuli to different and nearby tissue areas is hard to achieve; and (vii) these solutions are unable to perform controlled time-dependent trajectories of the bone formation process.

To date, only instrumented passive systems have been implanted in human patients^8^,^9^. Their operations have been restricted to measure biomechanical and thermodynamic quantities (forces, moments, deformations and temperatures) *in vivo*^23^,^24^,^25^. Instrumented implants that can ultimately control bone regrowth are highly desirable, particularly when designed with inner monitoring and delivery system functionalities. To this end, efforts have been focused on the development of biophysical stimulation systems, to exploit the potential of organic adaptation to exogenous excitations. Encouraging results have been observed *in vivo* using mechanical stimulation driven by piezoelectric actuators^26^. However, there is an increased risk of weakening the bone-implant interface bonding if the stimulators are located in the implants’ surface. In addition, no technological solutions using stimulators housed inside implants to stimulate target tissues were found. Ultrasound stimulation is hard to achieve when using instrumented implants. Such implants tend to have complex designs and may require hollowed structures that increase implant fracture risks. When intramedullary implants were modified to deliver magnetic field (MF) stimulation induced by permanent magnets, a notable bone formation was observed around the implant^27^. However, a major drawback of these implants is their inability to perform controllable and personalized tissue responses, since they only provide static stimuli^27^. To overcome these limitations, electromagnetic-stimulating hip systems have been proposed to enable the application of controllable electric field (EF) stimulation^28^. However, this solution is not able to deliver different stimuli to very close targets, and thus unable to geographically control the evolution of the bone formation process. This limitation is due to the following: (i) the stimulators’ electrodes are attached to the implant’s surface; and (ii) the electrical fields are obtained through extracorporeally-induced electromagnetic fields. Indeed, the current clinical practice that uses biophysical stimulation for bone remodeling is focused on delivering extracorporeal stimuli. This method does not concentrate the stimuli density over the bone-implant interface, as desired; rather, it is focused mainly on tissues that surround the skin. Another method for delivering EF/electromagnetic (EMF) stimulation, with demonstrated *in vitro* osteogenic-like responses^29^,^30^, is the capacitive coupling (CC) stimulation. Up-regulatory effects on various cellular mechanisms that underlie bone formation can be realized using CC stimulators to deliver stimuli to osteoblasts *in vitro* (cell lines, stem cells and primary cells)^31^,^32^,^33^,^34^. Extracorporeally-induced CC stimulation has also been used in clinical trials; its ability to improve the treatment of nonunions and osteoarthritis, as well as to minimize post-treatment complications, has been reported^29^. Nevertheless, the most common CC architecture uses electrodes in parallel, as shown in Fig. 1. These cannot be integrated into instrumented implants because the resulting stepped implant’s surface would most likely hinder bone-implant integration.

Significant advances in the scope of active implants will be achieved if solutions for long-term, highly controllable and personalized bone-implant bonding are developed. Here, a novel therapeutic delivery system with the potential to meet these demands is proposed.

## The concept of cosurface-based capacitive delivery system

Optimized osteogenic responses can be achieved if the stimulation systems fulfil the following requirements:

- Deliver non-cytotoxic and non-genotoxic stimuli;
- Enable the delivery of controllable stimuli, such that different stimuli (varying waveform, strength, frequency, periodicity, stimulation exposure, etc.) can be applied to various regions of target tissue;
- Allow programmed time-dependent stimulation for personalized medicine;
- Ensure everlasting operation throughout the patients’ lifetime, such that revision procedures can be avoided;
- Enable the application of therapeutic stimuli according to the osseointegration states;
- Allow miniaturized, stretchable and flexible integration inside the implant system.

**Figure 2:**
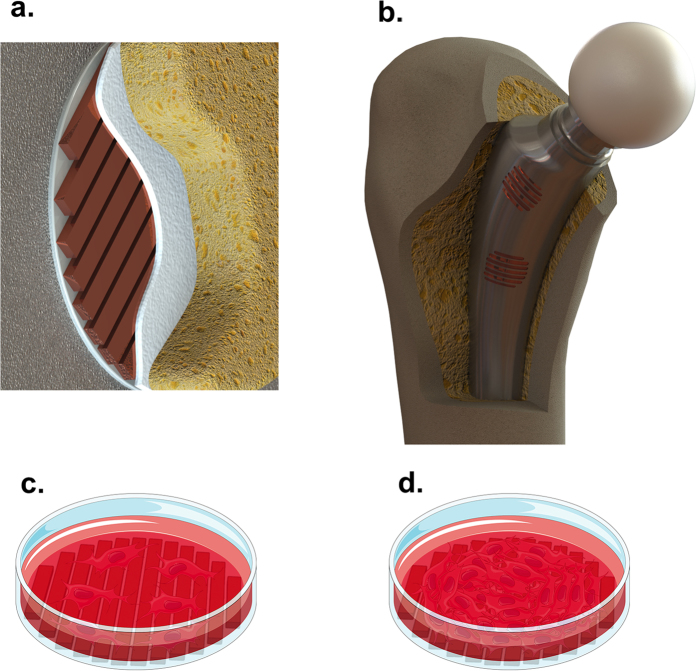
Cosurface-based CC stimulators according to a striped pattern. **(a)** Embedded for operating inside implant systems. (**b)** Example of instrumented hip prosthesis with ability to control osteoregeneration using these stimulators. (**c)** Apparatus for analysing the osteogenic results *in vitro* in the pre-confluent cell culture. (**d)** Apparatus for analysing the osteogenic results *in vitro* in the confluent cell culture.

To our best knowledge, current methodologies in this field are not feasible for effective bone remodeling. To address this deficiency, we propose a new concept for delivering EF stimuli, the ‘cosurface-based CC delivery system’, that satisfies the six requirements (a–f). This is a non-complex and cost-effective system, with a non-parallel architecture, capable of delivering EF stimuli using electrodes in the same surface, regardless of its topology. It can comprise as many electrodes as required that can function independently, allowing the delivery of EF stimuli to tissue areas in both a quasi-homogeneous and a heterogeneous manner. Furthermore, the electrodes can be shaped with arbitrary geometries and separated from adjacent electrodes by variable gaps. The solution presented here is in the form of a stimulator composed by a set of identical stripe-shaped electrodes, separated from each other by identical gaps, as shown in Fig. 2.

Our strategy to fulfil the requirements (a–f) above is as follows. EFs and EMFs do not introduce toxic chemicals and do not induce immunogenic responses to foreign bodies^35^, although autophagy is significantly induced by very high EFs. Furthermore, most of the studies that have analyzed the potential genotoxic effects of EFs/EMFs have not reported damage to the genetic material^36^. Hence, requirement (a) will most likely be fulfilled if the field strength-dependent toxicity of the delivered stimulus is taken into consideration.

Arbitrary and time-dependent excitations can be applied to each pair of electrodes, including different waveforms (sinusoidal, square, triangular or sawtooth, as well as arbitrary periodic or even non-periodic ones), amplitudes, frequencies, periodicities, daily stimulation exposures, resting time and total therapy duration. Various regions of a target tissue can be differentially stimulated by defining the most appropriated locations to embed the stimulators inside implants, by choosing optimized stimuli parameters for each region, as well as by optimizing the cosurface architecture. Numerical field analysis can be used to predict EFs/EMFs that are delivered to the peri-implant bone volume, as a function of imposed excitations to electrodes, as well as to optimize the stimulators’ design in advance of the implant’s body insertion. In addition, homogeneity of the delivered EF/EMF can be controlled by choosing which sets of electrode pairs will be delivering stimuli to an area(s) of the tissue. All these degrees of freedom provide the ability to produce customized stimuli for personalizing the administration of either prophylactic or corrective therapies. The stimuli controllability can also be increased since its delivery can be made controlled by clinicians through the use of wireless communication between the implant and extracorporeal systems^16^. Using these approaches, this new therapeutic design is able to comply with requirements (b) and (c) above.

An everlasting operation, as required in (d), can be accomplished by designing self-powered instrumented implants. Osseointegration states must be monitored for optimal performance of the implants^16^. Measurement operations can be performed by the instrumented active implants (via monitoring systems contained within the implants) and/or extracorporeally (via imaging techniques)^16^. These monitoring systems can be used to satisfy requirement (e), and will establish a feedback control over the stimuli delivery.

Stimulators must be positioned inside the implant, close to the implant’s surface, and a biocompatible coating must be used to ensure an absolutely safe encapsulation, as highlighted in Fig. 2a. The conformal integration of these stimulators with instrumented implants, in a way that it withstands mechanical deformations and satisfies requirement (f), is already feasible due to the current advances in materials, mechanics, and manufacturing engineering^37^.

As an example of the application of this concept, the stimulators can be embedded in the proximomedial region of instrumented active hip prostheses, as illustrated by Fig. 2b. In this type of prosthesis, the stress distribution is usually reduced following arthroplasty, which can result in bone loss and consequent implant failure^13^. Notice that although osseointegrative implantable devices may exhibit complex outer surfaces and electrodes can be arranged along similar surfaces (inside implants), the stimulators’ architecture can be design to lie along other surfaces (including the coplanar arrangement), which may be required to optimize the stimuli controllability. This new concept can also be used for other intracorporeal implants and to stimulate many other tissues.

## Results

This study is multifaceted and includes the introduction of a novel cosurface architecture for CC stimulators, the use of numerical models to predict the EFs/EMFs delivered by the stimulators to cell cultures, and the first osteogenic *in vitro* responses obtained using such an apparatus. A stimulation apparatus that highlights the potential of this new approach was demonstrated by providing a macro-scale system that mimics the stimulators embedded within active implants, close to the surface of the implant, as shown in Fig. 2c,d and described in the Methods section. Biological *in vitro* experiments using MC3T3 cells were carried out to analyze the three principal stages of bone remodeling (cellular proliferation, matrix maturation and matrix mineralization)^38^. Cosurface CC stimulators were driven by two distinct time-dependent excitations defined as K_EX_ (1 − cos(*w*t)) V, where *w* is the excitation angular frequency, and according to the following parameters:

- **LF EX**: K_EX_ = 5 (peak-to-peak amplitude of 10 V), 14 Hz frequency, 4 h/day exposure, unto 21 days of exposure;
- **HF EX**: K_EX_ = 5, 60 kHz frequency, 0.5 h/day exposure, unto 21 days of exposure.

These excitations have been chosen as reported in the Methods section. Charged electrodes of the same polarity were separated from one another (with anodes being always surrounded by cathodes). The spatially distributed EF and MF strengths were identified when delivered to:

- A plate with a pre-confluent cell culture (low cell confluence condition), which is considered as an approximation of two homogeneous phases, one above the other (Fig. 2c). This comprises a cellular layer (10 μm thick), which is adherent to the bottom surface of petri dishes due to the high percentage of adhesion of MC3T3 cells to polystyrene surfaces^39^; wherein this layer is covered by the cell culture medium (i.e., a liquid solution 1 mm thick);
- A confluent cell culture (full cell confluence condition) (Fig. 2d) similar to the low cell confluence condition, but now assuming that the adherent organic layer resembles an organized cellular tissue of 20 μm thickness, composed by MC3T3 cells and type-I collagen (‘collagen-I’), since this protein corresponds to ≈90% of the bone organic matrix.

Such analyses were carried out to characterize how cell cultures are stimulated throughout proliferation (mainly in pre-confluent cultures) and differentiation (mainly in confluent cultures) stages, as well as to study the influence of the dielectric properties of each phase (air, organic, liquid) on the stimuli delivery. Maxwell’s equations that govern the dynamics of EFs and MFs were solved using numerical modeling (as described in the Methods section). It is noteworthy that a model to predict EFs and MFs that stimulate cells during the matrix mineralization stage was not developed in this study.

### *In-silico* experiments

**Figure 3:**
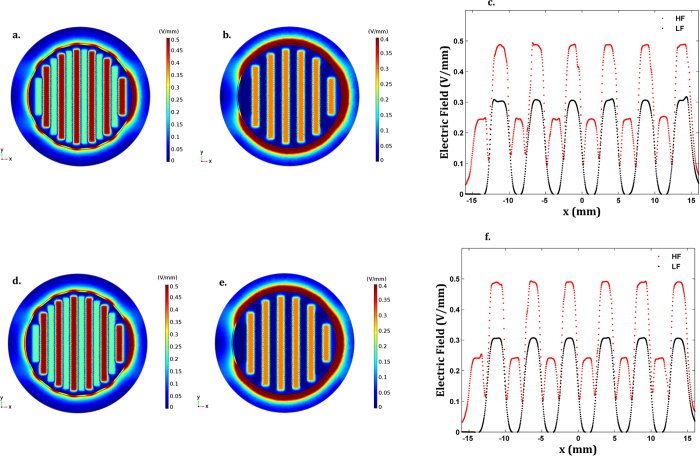
Simulation results of EF distributions and strengths. (**a,b**) 2D EFs stimulating cells at π rad when using low cell confluence condition, z = 0.505 mm and HF ST or LF ST, respectively; (**c)** EFs stimulating cells at π rad along the *x*-axis in the (**a,b)** conditions. (**d,e)** 2D EFs stimulating cells at π rad when using full cell confluence condition, z = 0.51 mm and HF ST or LF ST, respectively; (**f)** EFs stimulating cells at π rad along the *x*-axis in the (**d,e)** conditions.

**Figure 4:**
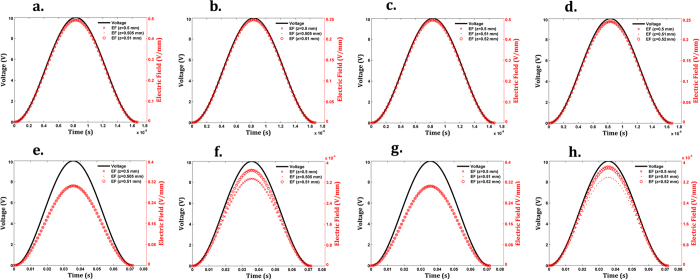
Simulation results of EF dynamics. **(a)** EFs in [0 2π] rad: HF ST at (x, y, x) = (−1.25, 0, 0.5005) mm, (x, y, x) = (−1.25, 0, 0.505) mm and (x, y, x) = (−1.25, 0, 0.5095) mm, low cell confluence condition; (**b)** EFs in [0 2π] rad: HF ST at (x, y, x) = (1.25, 0, 0.5005) mm, (x, y, x) = (1.25, 0, 0.505) mm and (x, y, x) = (1.25, 0, 0.5095) mm, low cell confluence condition; (**c).** EFs in [0 2π] rad: HF ST at (x, y, x) = (−1.25, 0, 0.5005) mm, (x, y, x) = (−1.25, 0, 0.51) mm and (x, y, x) = (−1.25, 0, 0.5195) mm, full cell confluence condition; (**d).** EFs in [0 2π] rad: HF ST at (x, y, x) = (1.25, 0, 0.5005) mm, (x, y, x) = (1.25, 0, 0.51) mm and (x, y, x) = (1.25, 0, 0.5195) mm, full cell confluence condition; (**e).** EFs in [0 2π] rad: LF ST at (x, y, x) = (−1.25, 0, 0.5005) mm, (x, y, x) = (−1.25, 0, 0.505) mm and (x, y, x) = (−1.25, 0, 0.5095) mm, low cell confluence condition; (**f).** EFs in [0 2π] rad: LF ST at (x, y, x) = (1.25, 0, 0.5005) mm, (x, y, x) = (1.25, 0, 0.505) mm and (x, y, x) = (1.25, 0, 0.5095) mm, low cell confluence condition; (**g).** EFs in [0 2π] rad: LF ST at (x, y, x) = (−1.25, 0, 0.5005) mm, (x, y, x) = (−1.25, 0, 0.51) mm and (x, y, x) = (−1.25, 0, 0.5195) mm, full cell confluence condition; (**h).** EFs in [0 2π] rad: LF ST at (x, y, x) = (1.25, 0, 0.5005) mm, (x, y, x) = (1.25, 0, 0.51) mm and (x, y, x) = (1.25, 0, 0.5195) mm, full cell confluence condition. (x, y) = (−1.25, 0) mm and (x, y) = (1.25, 0) mm are midpoints above the central electrodes.

Throughout the proliferation stage (low cell confluence condition), frequency-dependent EF patterns are observed. The highest EFs on the cellular layer (in the range z ε [0.5 0.51] mm above electrodes) are situated above the electrodes when the HF EX is applied: approximately 0.5 V/mm above the positively polarized electrodes (at the midpoints x = −11.25, −6.25, −1.25, 3.75, 8.75, 13.75 mm) at π radians of the HF EX, and 0.25 V/mm above the negatively polarized electrodes (at midpoints x = −13.75, −8.75, −3.75, 1.25, 6.25, 11,25 mm) at the same time instant (Fig. 3a,c and Supplementary Fig. S1a,e). EF strengths decreasing up to 0.1 V/mm above the gaps should also be noted. The EF dynamics is quite similar to the excitation dynamics, as depicted from the cross-correlations of nearly 100% between the generated EF waveforms and the excitation waveforms (Fig. 4a,b and Supplementary Fig. S2a,b,e,f,i,j,m,n,q,r). The cellular layer was stimulated with different EF strengths when electrodes were excited with LF EX. The highest EFs are concentrated above the positively polarized electrodes (fields approximately 0.3 V/mm), which decrease to low strengths (always below 8 × 10^−4 ^V/mm) as the distance to the negatively polarized electrodes decreases, as shown by Fig. 3b,c, Supplementary Fig. S1d,f, and Fig. S2d,h,l,p,t. EFs above the gaps present higher strengths than those above the negatively polarized electrodes, but they are close to those found using HF EX. Similar cross-correlations between excitations and generated EFs can be identified in comparison with results obtained for HF EX (Fig. 4e,f and Supplementary Fig. S2c,d,g,h,k,l,o,p,s,t). The MF strengths are lower than the lowest osteogenic magnetic fields reported in the literature (either at low or high frequency)^40^, and will most likely induce negligible osteogenic effects.

Patterns and strengths of the stimuli delivered to the cellular tissue (in the range z ε [0.5 0.52] mm above electrodes) throughout matrix formation and maturation (full cell confluence condition) are similar to those delivered during cell proliferation (Figs 3d–f and 4c,d,g,h and Supplementary Fig. S1c,d,g,h). MFs stimulating the cellular tissue also present similar strengths as the ones observed for the low cell confluence condition and, consequently, will most likely not be osteogenic. Hence, two time-dependent EF stimuli, also defined as K_EF_ (1 − cos(*w*t)) V/mm (where *w* is the stimulus angular frequency), were approximately delivered to cells both during the pre-confluency and confluency stages, according to the following parameters:

- **HF ST**:

- Above positively polarized electrodes: K_EF_ ≈ 0.25, 60 kHz frequency, 0.5 h/day exposure, unto 21 days of exposure.
- Above negatively polarized electrodes: K_EF_ ≈ 0.125, 60 kHz frequency, 0.5 h/day exposure, unto 21 days of exposure.
- Above gaps: K_EF_ ≈ 0.05, 60 kHz frequency, 0.5 h/day exposure, unto 21 days of exposure.

- **LF ST**:

- Above positively polarized electrodes: K_EF_ ≈ 0.15, 14 Hz frequency, 0.5 h/day exposure, unto 21 days of exposure.
- Above negatively polarized electrodes: K_EF_ ≈ 0.2 × 10^−4^, 14 Hz frequency, 0.5 h/day exposure, unto 21 days of exposure.
- Above gaps: K_EF_ ≈ 0.05, 14 Hz frequency, 0.5 h/day exposure, unto 21 days of exposure.

LF ST and HF ST will be referred hereafter as the biophysical stimuli delivered during osteoblast proliferation and differentiation stages. These results highlight a well-defined EF distribution and dynamics along the cellular layer(s). Although only the LF ST and HF ST were biologically tested in this work, the ability of the cosurface stimulator to deliver other required stimuli to target regions during the first two stages of bone remodeling is also demonstrated. Supplementary Figs S3 to S9 provide a selected set of simulation results that highlight the ability of the cosurface stimulator to deliver controllable stimuli to target regions through the pre-confluent or full confluent cell cultures. Required EF strengths can be obtained by defining proper excitation amplitudes, as field strengths are linearly related to the excitation amplitudes (Supplementary Fig. S3). EF heterogeneity can be controlled by changing the excitation of each electrode, as well as by modifying the geometry of the stimulator. As shown in Supplementary Fig. S4a,b,f, EF homogeneity can be maximized by positively polarizing all electrodes, except those in the end extremities, and minimizing the gap width. Different EF heterogeneities can be obtained using different polarizations, such as defining different K_EX_ for each electrode while keeping anodes always surrounded by cathodes (Supplementary Fig. S4c–f), and using different width of gaps and electrodes (Supplementary Fig. S5a,b). Smooth EF distributions can be obtained by rounding the electrodes’ corners (Supplementary Fig. S5a,b). Further, EFs with a required waveform, strength and frequency can be delivered to the cellular layer/tissue (Supplementary Fig. S6a–l). Moreover, different and nearby tissue areas can be simultaneously and differently stimulated by supplying electrodes with different excitations, as illustrated by Supplementary Fig. S4c–f. We can then infer from these results that this cosurface capacitive stimulator enables the delivery of controllable EF stimuli throughout the two first stages of bone remodeling. Interestingly, due to the dielectric and magnetic interactions among the different domains, a fall in the EF strengths is not always verified along the *z*-axis of the cellular layer/tissue with increasing distance to the electrodes. Instead, spatially-dependent tri-dimensional patterns define these EF strengths, as highlighted by Supplementary Figs S7 and S8. It should however be noted that differences along the *z*-axis are minute (Fig. 4f,h and Supplementary Fig. S9).

### *In-vitro* experiments

**Figure 5:**
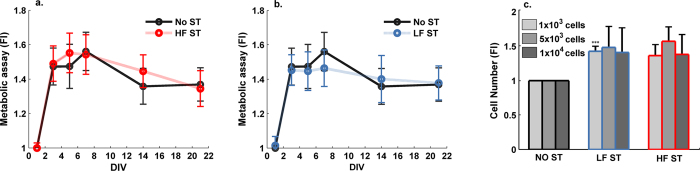
Influence of the LF ST and HF ST on the number of viable pre-osteoblastic cells. MC3T3 cells were seeded at 1.8 × 10^4^ cells/cm^2^ and daily exposed to: (**a)** NO ST and HF ST or (**b)** NO ST and LF ST. The number of viable cells was indirectly accessed by the metabolic reversible resazurin assays at the indicated days *in vitro* (DIV) (n = 6–8). Statistically significant differences of metabolic activity were found throughout cell culture (ε = 0.395; F_GG_(1.975,23.705) = 27.786; p < 0.001; 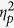 = 0.698), 1 DIV vs 3, 5, 7, 14, 21 DIV and 4 DIV vs 6 DIV p < 0.001. No statistically significant differences between groups (F(2,12) = 0.433; p = 0.658; 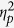 = 0.067). (**c)** The number of viable cells was directly scored 24 h upon seeding the MC3T3 cells at increasing cell densities: 1 × 10^3^, 5 × 10^3^ and 10 × 10^3^ cells/cm^2^. Cell number was scored using Trypan blue, a membrane exclusion dye (n = 7–15). All data are presented as fold increases (FI) over NO ST levels at 1 DIV. Statistically significant differences between groups in 1 × 10^3^ cells/cm^2^ cell density: F(2,29) = 5.772; p = 0.008; 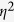 = 0.284; ***p < 0.001 for NO ST vs LF ST (Games-Howell post-hoc analysis).

The biological effects of these stimuli on the viability and proliferation of the pre-osteoblastic MC3T3 cells were analyzed. The non-toxic resazurin method was used to monitor cell viability with time in culture. A total of 1.8 × 10^4^ cells/cm^2^ cells were plated and followed for various days *in vitro* (DIV) (Fig. 5a,b). Since no significant differences were observed in the initial cell density, and major alterations in cell proliferation usually occur at the pre-confluent period, lower initial cell densities were subsequently tested (1 × 10^3^, 5 × 10^3^ and 10 × 10^3^ cells/cm^2^). In this test, cells were directly scored after 1 DIV (24 h), using an exclusion dye (Fig. 5c). Both LF ST and HF ST tended to increase the cell number in these latter conditions, but more consistently and significantly at the lowest cell density (1 × 10^3^ cells/cm^2^) (Fig. 5c).

**Figure 6:**
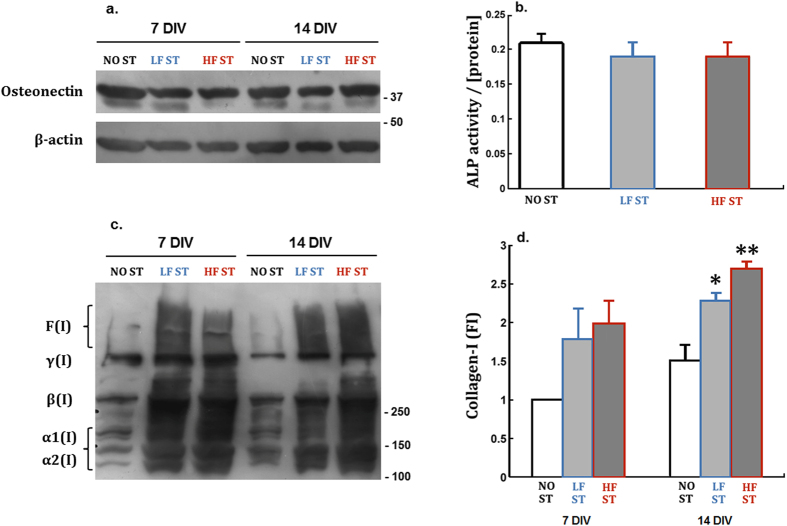
Relative expression and activity of three matrix maturation protein markers. **(a**) Immunoblot analysis of the osteonectin expression in MC3T3 cells exposed for 7 and 14 DIV to NO ST, LF ST, and HF ST. β-actin was used as loading control; no differences were observed in between the experimental conditions. (**b)** The intracellular ALP activity was quantified in 1.8 × 10^4^ cells/cm^2^ MC3T3 cells daily exposed to the LF ST and HF ST for 15 DIV. ALP activity values, determined by measuring the conversion of the ALP substrate (p-Nitrophenyl phosphate) at 405 nm by cells lysates, were divided by the total protein concentration of each lysate. No statistical significant differences between groups (F(2,15) = 0.604; p = 0.56; 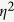 = 0.074). (**c,d)** The expression of cell-associated collagen-I was analyzed by immunobloting the lysates of LF ST and HF ST stimulated cells (**c**) and the levels of collagen-I bands quantified for each condition (**d**). Collagen-I forms: unprocessed and processed α1(I) and α2(I) procollagen monomeric chains (130–160 kDa); β(I), procollagen dimeric forms (≈270 kDa); γ(I), procollagen trimeric forms (≈400 kDa); F(I), collagen fibrils. No significant differences between groups at 7 DIV (F(2,6) = 2.929; p = 0.13; 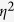 = 0.494). Statistically significant differences between groups at 14 DIV (F(2,6) = 17.543; p = 0.003; 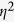 = 0.854); *p < 0.05 for NO ST vs LF ST (p = 0.027); **p < 0.01 for NO ST vs HF ST (p = 0.003) (Bonferroni post-hoc analysis).

Biological outcomes resulting from delivering the LF and HF stimuli during the differentiation stage were evaluated by monitoring the production and secretion of collagen-I, and of a noncollagenous protein (osteonectin). These are widely used early biomarkers of osteoblastic differentiation, together with the activity of the alkaline phosphatase (ALP) enzyme. When analyzing the cellular (Fig. 6a) and secreted (Supplementar Fig. S10) levels of osteonectin, no differences were detected between the control cells (NO ST, ‘no stimulus’) and cells under LF/HF stimuli at various DIV. When assaying for cellular ALP activity at 15 DIV, a time period of matrix maturation, again no significant differences were detected (Fig. 6b). However, when collagen-I levels were evaluated and quantified in immunoblot assays, a major increase (1.8–2.7 times) was observed at 7 DIV and particularly at 14 DIV, for cells daily exposed to the LF and HF stimuli (Fig. 6c,d). All procollagen forms, from α monomers, β dimers, γ trimers, to collagen fibers were elevated. The levels of secreted procollagen were also monitored, and at 14 DIV the LF and HF stimuli also tended to increase procollagen secretion (Supplementary Fig. S10).

**Figure 7:**
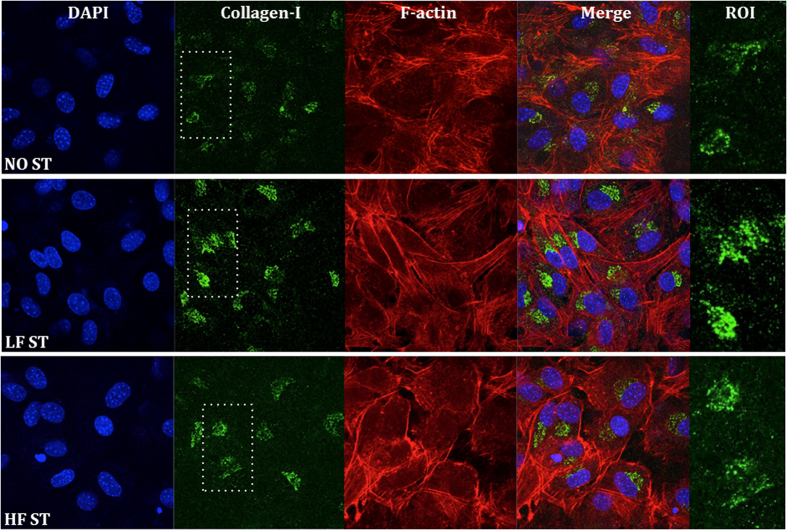
Confocal microscopy analysis of intracellular collagen-I in MC3T3 cells exposed to LF ST and HF ST for 21 DIV. Fixed cells were subjected to immunocytochemistry procedures in order to detect collagen-I distribution (green fluorescence). The cytoskeleton constituent filamentous actin (F-actin; labeled with red fluorescing phalloidin) and nucleic acids (labeled with blue fluorescing DAPI) were used as cellular counterstaining. Bar, 10 μm. ROI is the region of interest of the green (collagen-I) channel (zoomed 2x).

The distributions of cells and collagen-I in the osteoblastic population were analyzed by confocal microscopy at 21 DIV. This is a period usually associated with matrix maturation and the onset of matrix mineralization. Intracellular and matrix collagen-I was immunostained (in green); filamentous actin (F-actin, in red) was detected to evidence the cellular contours and cell-to-cell contacts (Fig. 7). Stimulation of cells by cosurface stimulators appeared to induce a more organized cellular tissue, with better-defined cell-to-cell contacts (Fig. 7, F-actin staining). Daily stimulation with the LF and HF stimuli for 21 DIV induced a higher content of collagen-I than control conditions. This is visible not only inside the cells but also at the extracellular matrix (dotted staining in Fig. 7 zoomed-in ROIs). At this period, the highest intensity of the matrix collagen appeared to result from LF stimulation.

**Figure 8:**
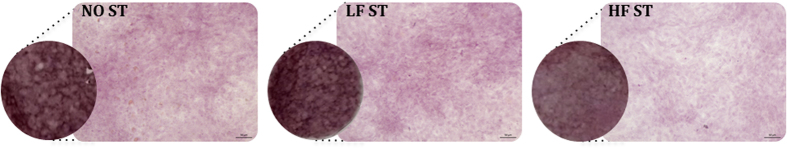
Visual analysis of the matrix mineralization in MC3T3 cells exposed to EMFs generated by LF EX and HF EX for 21 DIV. Fixed cells in 35 mm culture plates were subjected to cytochemistry procedures with Alizarin Red, which marks calcified extracellular matrices. Photographs of representative Alizarin Red-stained circular plates are shown, together with zoomed in insets taken with a light microscope under a 10x objective (right rectangles). Bar, 50 μm.

Since a recently developed organic matrix needs to be mineralized to generate a functional mature bone matrix (stage three), the status of matrix mineralization was further assessed. Alizarin Red, a dye that stains calcified extracellular matrices, was used. In Fig. 8, photographs of representative Alizarin Red-stained circular plates and zoomed-in microphotographic insets reveal that the different frequencies had opposite effects on matrix mineralization. While the LF stimulus tended to maintain or even slightly increase matrix mineralization, daily stimulation with the high frequency stimulus led to a decrease in cellular mineralization (Fig. 8).

## Discussion

Stimulation of osseointegration is an essential capability of implants^10^. Although the primary stability following implantation is obtained by mechanical press-fit, secondary stability requires optimized bone remodeling, both on host bone and implant surfaces (distance and contact osteogenesis)^10^. Control of the peri-prosthetic bone stock is mandatory to avoid surgical revisions caused by adverse bone remodeling that can occur after implant insertion^10^,^13^. In this paper, an initial evaluation of the potential of delivering EMF stimuli via cosurface CC stimulators to control osseointegration was performed, and their effectiveness in delivering desirable EF stimuli to target regions, together with their osteogenic-like properties, were monitored. The preliminary testing, using only two stimuli, demonstrated promising results on the capability of the cosurface stimulators to induce positive osteogenic responses at different stages of bone remodeling. The cosurface stimulation apparatus was effective in inducing cellular proliferation at early time points and when the culture was at low cell densities. Subsequently, both LF and HF stimuli highly induced cellular collagen-I at the matrix maturation period (7–21 DIV), potentiating matrix maturation (21 DIV). Regarding the third stage of osteogenesis, this CC architecture can maintain or even slightly increase matrix mineralization (delivering the LF stimuli), or can postpone it (delivering the HF stimuli). The success obtained in terms of osteoblastic proliferation and differentiation provides strong motivation and justification for further development and application of cosurface stimulators in personalized biophysical-based therapies for bone-implant integration. This requires further research to identify the interplays between the stimuli parameters and their osteogenic and osteoinductive properties. More systematic studies must be conducted, analyzing how bone cell model systems (as immortalized lines, stem cells, and primary cultures) respond to the various stimuli generated by the cosurface architectures. Biological readouts should include cell proliferation, production of structural and regulatory matrix proteins (collagen-I, fibronectin, alkaline phosphatase, etc.), and matrix maturation and mineralization markers (as osteopontin, osteocalcin, bone sialoprotein, hydroxyapatite mineral crystals, etc.). An extensive understanding of these cellular responses to EFs and MFs is essential for optimizing stimuli parameters to be used in therapeutic stimulation of the bone tissue.

This study provides for the first time an analysis of cosurface CC stimulation when delivering EFs and MFs to bone-related cells. Since this cosurface CC stimulation produces more complex dynamics than parallel CC stimulation, we have considered less complex models in our preliminary studies that nevertheless provide reasonable predictions of how MC3T3 cells are stimulated by EFs and MFs. We made the following assumptions in our analysis: (1) during the proliferation stage, the cellular phase is approximately homogeneous and mainly composed by MC3T3 cells; (2) the cellular tissue formed after cell confluency and before matrix mineralization is also nearly homogeneous and mainly composed by MC3T3 cells and collagen-I; and (3) the dielectric properties of MC3T3 cells and hydrated collagen are similar. Undoubtedly, further research efforts must be conducted to develop more complex models. Nevertheless, under the conditions established here to carry out the stimulation tests, minor influences of EFs and MFs are expected as a result of: (i) the low concentration of the physiological culture medium around cells; (ii) the time-dependent dielectric properties of the cellular medium due to dynamics associated to the proliferation and differentiation stages; and (iii) the heterogeneity of the different phases, among others. However, more complex dielectric structures will most likely be observed as higher concentrations of the inorganic components of the bone matrix are extracellularly deposited, which will require the development of more complex models. The use of numerical models is also envisaged in future research in order to investigate the capability of the cosurface stimulator to deliver desirable EFs to target regions of cancellous bone tissue. Actually, the inhomogeneity of trabecular structures resulting from their liquid content and crystalline and amorphous mineral and organic phases must be accurately modeled. Numerical analysis can also enable the geometric optimization of cosurface architectures, such that required surface shapes can be designed to optimize stimuli parameters such as EF strengths, directions, homogeneity, etc. The final aim is to embed these novel cosurface CC stimulators into self-powered instrumented active implants for controlling the peri-implant bone volume. Such methodology may achieve a superior implant performance due to its potential for tracking optimized spatio-temporal trajectories of osteoblasts proliferation, matrix maturation and mineralization^16^. These future implants will ultimately be controlled by a clinician. Besides the innovations carried out to design an efficient therapeutic delivery system, which is the subject of this paper, other significant technological advances have been achieved that optimize other systems required to the full operation of instrumented active implants, namely their communication, monitoring and powering systems. Telemetric systems have been developed for establishing communication between instrumented implants and extracorporeal systems^8^,^23^,^24^,^25^. This communication can be optimized for monitoring the osseointegration status and establishing a therapeutic stimulation based on the decisions of the clinician. Instrumented implants can thus be designed as slave systems commanded by master systems located outside the patients’ body, allowing to define and modify time-dependent therapeutic commands^16^. Since sophisticated communication systems have already been developed for instrumented implants^8^,^23^,^24^,^25^, the addition of these new features is not envisaged as complex. Hence, the stimuli parameters, such as field strength, frequency, periodicity, resting time, stimulation time, field homogeneity, among others, can be changed after implant insertion and according to the patients’ idiosyncrasies.

The self-powering capability of these electrostimulative implantable devices is crucial for long-term stimuli delivery. Such capability can enable a superior implant performance as the stimuli can be delivered autonomously throughout the routine activities of the patients^16^. Motion-driven electromagnetic energy harvesting has been suggested as the most promising method to power these implants, as they avoid adverse effects on the bone-implant fixation^8^,^41^,^42^,^43^. Harvester architectures that generate electrical power using motions of levitated hard-magnetic elements have been the main ones explored for such goal, since their designs are non-complex yet customizable under severe dimensional constraints, as well as due to their inherent maintenance-free operation and long-term stable performance^41^,^42^,^43^. Significant research efforts have been conducted to evaluate the maximum energy they are able to harvest, according to the dimensional constraints imposed by implants and the gait patterns of patients. The development of nonlinear models of their transduction mechanisms has been carried out for design optimization prior to fabrication, location optimization, and adaptability requirements^42^. The effectiveness of these harvesters will be ascertained after this optimization process, including the ability of these harvesters to deliver voltages up to 10 V. This does not exclude the analysis of the osteogenic and osteoinductive effects due to lower EFs, obtained with much lower voltage excitations. Nevertheless, voltage levels up to 10 V may be achieved by charging storage systems (recharging batteries and/or supercapacitors). An integrated solution, that may include storage and power management systems, will most likely be required, mainly to ensure electric power supply during the perioperative period, in old ages and when demanding therapies are required (e.g. several hours of daily stimulation). These have also been optimized for such purpose^43^.

In parallel, some breakthroughs mainly involving acoustic-mechanical methods and motion-driven piezoelectric sensing^8^, have appeared and will support the development of instrumented implants capable of aseptic loosening detection. Nevertheless, more complex measurement systems must be designed to monitor the osseointegration states required for supporting effective administration of biophysical therapies. To find optimized trajectories of osteoblast proliferation, matrix formation, maturation and mineralization, the bone-implant interface must be characterized, in particular along the critical regions (such as the proximomedial region of hip prostheses)^16^. Potential measurable states are the rate of cell adhesion to the implants’ surface, and the mechanical behavior and topological characterization of the bone-implant interface, among others. Should not be dismissed that invasive methods used to track the osseointegration states may produce adverse effects on the bone-implant fixation. To ensure long-term operation of active implants, the most promising approach seems to be the development of miniaturized and stretchable monitoring systems to be embedded into the implants. These must be located as close as possible to the stimulatory systems and allow a controllable time-dependent operation defined by clinicians.

As these instrumented implants demand inner hollowed structures, their mechanical stability must be carefully considered. Instrumented joint replacements were already implanted in dozens of patients, which have been accomplished after many successful tests conducted to analyze their mechanical stability^8^,^9^, and more than ten years of successful operation have been achieved^8^,^9^,^23^,^24^,^25^. It should also be noted that their powering, monitoring, communication and data processing systems have been housed in different locations. To date, no significant differing mechanical properties, which could considerably increase risks of implant fracture or weak bone-implant integration, have been reported. The addition of small-scale cosurface-based capacitive delivery systems into instrumented implants will most likely result in negligible effects on mechanical stability. Nevertheless, for reliable massive use of these implants, it is reasonable to develop advanced materials with improved bulk properties that can ensure the mechanical integrity of these hollowed implants.

Finally, electrodes of cosurface stimulators can now be projected to ensure outstanding electric conductivity properties under large mechanical deformations, which is highly desirable for designing stretchable and flexible cosurface stimulators^37^.

## Methods

### Cosurface stimulation apparatus

The cosurface-based CC stimulator is composed by 12 stripe-shaped electrodes that are 2 mm wide, 1 mm thick and of different lengths (2 × 12 mm, 2 × 20 mm, 2 × 25 mm, 2 × 28 mm, 2 × 30 mm, 2 × 31 mm). A 0.5 mm gap between electrodes was set. This geometry was designed to stimulate cell cultures on petri dishes of 35 mm in diameter by positioning these cell culture-containing dishes over the stimulators, which in turn are glued to a polymeric substrate. In this configuration, the petri dish thickness prohibits cell-electrode contacts. Electrodes were fabricated in copper due to their very high electrical conductivity. Stripes were machined by conventional technology. Polystyrene dishes and polycarbonate substrates were chosen due to their very high electrical resistivity. Thicknesses of 0.5 mm for polycarbonate substrates and polystyrene dishes were used.

Excitations to power stimulators were configured using a real-time application that was developed using Simulink (v. 7.3, Mathworks) and the Real Time Workshop (v. 7.3, Mathworks) and run using the Real Time Windows Target (v. 3.3, Mathworks) kernel. Excitations were generated by an IO card (MF 624, Humusoft).

### Numerical simulation

The finite element method was used to solve the constituent Maxwell’s equations that govern the dynamics of EFs and MFs. The finite element models were developed using the AC/DC module of COMSOL Multiphysics (v. 4.4, COMSOL). Each component of the models was considered homogeneous and isotropic. Each model was built using 17 domains: 12 electrodes, petri dish, substrate, cellular medium, physiological medium and air. Each domain ‘Electrode’ was modeled as a stripe with a wired connection in its normal direction. These ‘wires’ were created as cylinders making a hole in the substrate. The domain ‘air’ was defined around the other domains, except the 12 ‘wires’ that were also used as terminals to apply both LF and HF excitations. The dimensions of each domain are described in Table 1.

The dielectric properties of MC3T3 cells were considered as previously reported by Ozawa *et al*.^44^ and Wiesmann *et al*.^32^. Similar dielectric properties of MC3T3 cells and hydrated collagen-I were assumed based on studies conducted by Tomaselli and Shamos^45^,^46^. Electric conductivity and permittivity of the physiological medium were respectively established as reported by Pucihar *et al*.^47^ and Ozawa *et al*.^44^. The full list of dielectric and magnetic properties of materials and media are summarized in Table 1.

The finite elements were assembled by creating refined 3D meshes of tetrahedral linear elements of second order to tessellate the entire geometry of materials and media used to simulate the delivery of EFs and MFs. Meshes of models to simulate the low cell confluence condition and the full cell confluence condition were refined until convergence errors lower than 2% were achieved. Although the same tessellation method was used (the Delaunay method), a different mesh size was considered for each domain. A model with a mesh comprising 1,021,662 elements was developed to simulate the low cell confluence condition (681,455 elements required to tessellate the 10 μm cellular layer); 1,015,950 elements were used to simulate the full cell confluence condition (677,300 elements for the 20 μm cellular tissue). The interior boundaries only assume continuity, corresponding to a homogeneous Neumann condition. Terminal nodes were used to provide boundary conditions to 6 electrodes for applying the external HF and LF excitations. Ground nodes were defined for applying zero potential as boundary conditions to the other 6 electrodes. Electric Insulation was added to all external boundaries. The ‘Magnetic and Electric Fields’ (mef) was the COMSOL physics interface utilized to solve the Maxwell’s equations in the frequency-domain, according to Eqs (1) to (8), 2, 3, 4, 5, 6, 7, 8:


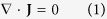



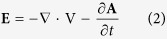



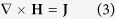



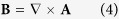



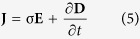



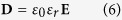



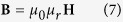



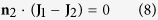


where **E** is the electric field intensity, **D** is the electric displacement, **H** is the magnetic field intensity, **B** is the magnetic flux density, **J** is the current density, **A** is the magnetic vector potential, ε_0_ is the permittivity of vacuum, ε_r_ is the relative permittivity, μ_0_ is the permeability of vacuum, μ_r_ is the relative permeability, σ is the electrical conductivity, V is the electric scalar potential and **n**_**2**_ is the outward normal from medium 2 at interfaces between two media (**J**_**1**_ and **J**_**2**_ are respectively the current densities of medium 1 and 2).

Simulations were carried out using the linear FGMRES (flexible generalized minimum residual) iterative solver (maximum number of iterations: 10,000) to avoid out of memory problems and to provide fast convergence and computing robustness. A fully coupled approach (automatic Newton method) was chosen (minimum damping factor: 1 × 10^−4^) since it can ensure convergence effectiveness because the initial conditions are well known (all electromagnetic quantities equal to zero), and the problem is differentiable. The dimensions of the ‘air’ domain were minimized while ensuring convergence errors lower than 2%. Models were computed in a workstation (Precision T5500, Dell) with 12 CPUs of 2.4 GHz and 24 GB RAM.

### Cell culture

The osteoblastic MC3T3-E1 cell line (ATCC, Barcelona, Spain; CRL-2593), established from C57BL/6 mouse calvaria, is widely used as a model for studying the various stages of osteogenesis *in vitro* and due to the similarity to primary calvaria osteoblasts^48^. Cells were maintained in a humidified atmosphere at 37 °C/5% CO_2_, in 2 mM glutamine-containing Minimum Essential α-Medium in Eagle’s balanced salt solution, supplemented with 10% (v/v) fetal bovine serum, 1% (v/v) of a 100 U.m/L penicillin and 100 mg.m/L streptomycin solution (Gibco BRL, Invitrogen, Life Technologies, Carlsbad, CA, USA), and 3.7 g/L NaHCO_3_. Cellular subcultures were performed using 0.05% trypsin/EDTA (Gibco BRL, Invitrogen). Stimulation of cells was carried out inside a CO_2_ incubator (Galaxy 14S, New Brunswick Scientific) featuring a communication port that was employed to deliver electric signals to electrodes.

### Excitations powering the cosurface stimulators

Stimulators were powered by excitations that were chosen on the following bases: the parameters chosen have resulted in osteogenic effects in *in vitro* studies using parallel CC stimulators; data from our pilot experiments were taken into consideration, as this capacitive stimulation method was never tested before; likewise, constraints imposed by instrumented active implants to electrically power the cosurface stimulators were also considered. To our best knowledge, the osteogenic effects of only three different stimuli, delivered *in vitro* to MC3T3 cells using CC stimulators, are reported in the literature^31^,^44^,^49^. These stimulation assays were nevertheless not designed to assess the three stages of osteogenesis, and only reported to enhance proliferation rates of these cells^31^,^44^. Among the EFs that have been used to stimulate bone cells *in vitro*, many have used EF strengths lower than 1 V/mm and electrode excitations up to 10 V, with at least one osteogenic stage of bone remodeling being found up-regulated in each study^31^,^34^,^38^,^49^,^50^,^51^. Much higher EF strengths (up to 21 V/mm) also enhance osteogenic processes, but very high voltages (hardly achievable by instrumented implants) are required to electrically supply the stimulators^33^,^41^. Hence, in our cosurface CC stimulator we applied voltages that generated EFs previously observed to be osteogenic for MC3T3 osteoblasts, considering the constraints above described. Frequency is among the main parameters that influence the osteogenic outcome^29^,^35^. While increased osteogenesis and osteoinduction has been reported mainly for low frequencies (lower than 16 Hz)^33^,^44^,^52^, it also occurs for high frequencies (60 kHz)^49^,^53^. Therefore, two frequencies within these ranges were analyzed using the cosurface CC stimulator. Given that a large number of studies on EF/EMF-induced bone cell stimulation have used sinusoidally-driven electrodes^30^,^31^,^49^,^50^, the same waveforms were used in our study. The daily exposures were based on other *in vitro* studies, where these parameters revealed to be osteogenic *in vitro*^38^,^51^,^54^,^55^.

### Resazurin metabolic and Trypan blue cell score assays

The resazurin colorimetric assay, which analyses the amounts of NADH/NADPH produced by metabolic active cells, was used to determine the effect of LF ST and HF ST on cell viability (as previously described^56^). Briefly, at 1, 3, 5, 7, 14 and 21 DIV, cells were incubated for 4 h with a 10% resazurin (Sigma-Aldrich) in MEM medium solution. Resazurin reduction was spectrophotometrically measured at 570 and 600 nm (Infinite M200 PRO, Tecan); the OD 570/OD 600 nm ratio was calculated for each condition^56^ and presented as fold increases over NO ST levels at 1 DIV. The trypan blue (Sigma-Aldrich) membrane exclusion assay was used in direct cell scoring to assess cell proliferation. Briefly, cells (1 × 10^3^, 5 × 10^3^, 1 × 10^4^ cells/cm^2^) were plated into 35 mm dishes (~10 cm^2^) with 1 mL fresh media. Upon 24 h in culture at 5% CO_2_/37 °C (either on the cosurface CC stimulators or not), the number of viable cells was scored (as previously reported^56^).

### Alkaline phosphatase activity

ALP is an enzyme that hydrolyzes organic phosphates to increase phosphate concentration and prepare the cell for matrix mineralization by hydroxyapatite crystals. Intracellular ALP activity was measured at 15 DIV using ALP substrate (p-Nitrophenyl phosphate, Merck Chemicals). Briefly, cells were washed with PBS, collected in Triton X-100 1% and incubated at 4 °C, 200 rpm. After sonication on ice (30 sec), the cellular content was homogenized and an aliquot (20 μl) transferred to a 96-well plate in triplicate. After incubation with 200 μl of the ALP substrate (1 h at 37 °C in dark), the enzymatic reaction was quantified in a microplate reader at 405 nm.

### Western blot analyses

At 7 and 14 DIV, cells and conditioned media were harvested into SDS 1% solutions and subjected to a 5–20% gradient (cells lysates) or 7.5% (cell media) SDS-PAGE and to western blot analyses (as previously described^57^). Previous to immunoblot, ponceau S reversible staining was first used for detection of total protein content on nitrocellulose membranes^58^. After overnight incubation at 4 °C with the primary antibodies (rabbit anti-collagen-I (1:1000); rabbit anti-osteonectin (1:500); mouse anti-β-Actin (1:1000), all from Novus Biologicals, Germany), horseradish peroxidase-linked (GE Healthcare, Chalfont St. Giles, UK) antibodies for enhanced chemiluminescence (ECL) detection were incubated 2 h/RT. Protein bands were scanned and quantified (GS-800™ Calibrated Densitometer and Quantity One densitometry software, Bio-Rad), and immunoblot data corrected to the respective ponceau loading control (as previously reported^58^).

### Morphological confocal microscopy analysis

MC3T3 cells grown on coverslips-containing 35 mm petri dishes and subjected for 21 DIV to LF and HF stimulation were fixed in 4% paraformaldehyde for 20 min and permeabilized with 0.1% Triton X-100 in PBS (15 min/RT). Immunocytochemistry of collagen-I, F-actin staining with phalloidin, and nuclear staining with DAPI was as previously described^59^. The anti-collagen-I antibody (1:250 in 3% BSA-PBS), a FITC-conjugated secondary antibody (1:300 in 3% BSA-PBS), and red fluorescing Alexa 568-labelled Phalloidin (1:500) were used. Images were acquired with a plan-Neofluor 40x/1.30 oil objective in a LSM 510 META confocal microscope (Zeiss, Jena, Germany) (as previously described^59^), in the iBiMED’s Imaging Facility, a node of PPBI (Portuguese Platform of BioImaging).

### Alizarin red staining visualization and quantification

Matrix mineralization was analyzed as previously described^60^. Briefly, cells were washed in PBS, fixed in 4% paraformaldehyde/20 min, washed in dH_2_O and incubated with Alizarin red solution (40 mM, pH4.1, Sigma; 20 min/RT). Non-incorporated staining was removed and cells thoroughly washed before image acquisition.

### Statistical analysis

The SPSS package v23 (IBM SPSS Statistics) was used to conduct all statistical analyses. All data is expressed as mean ± standard error of the mean. One-way ANOVA followed by post-hoc analysis were performed to compare biological data between groups (No ST vs LF ST vs HF ST). Mixed design factorial ANOVA was used to compare variability between and within (along various DIV) groups in the metabolic activity assay. All the requirements were previously verified using the following tests: Kolmogorov-Smirnov test and Shapiro-Wilk test for normal distribution assessment; Levene’s test and Sphericity analysis to assess homogeneity of variances and/or covariances. Eta squared (η^2^) and partial Eta squared (η^2^_p_) are expressed as measures of effect size.

## Additional Information

**How to cite this article**: Soares dos Santos, M. P. *et al*. New cosurface capacitive stimulators for the development of active osseointegrative implantable devices. *Sci. Rep*. **6**, 30231; doi: 10.1038/srep30231 (2016).

## Supplementary Material

Supplementary Information

## Figures and Tables

**Figure 1:**
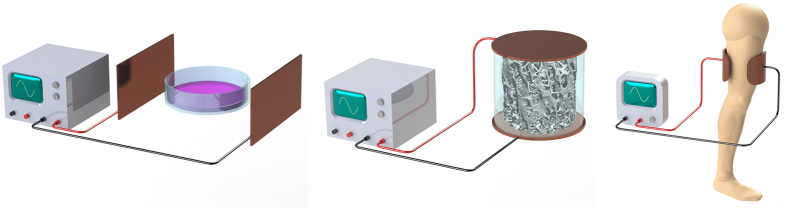
CC stimulation apparatus using electrodes in parallel configuration. (**a**) *In vitro* stimulation of bone cells. (**b**) *In vitro* stimulation of bone tissue. (**c**) Clinical usage of CC stimulation.

**Table 1:** Dimensions of domains, dielectric and magnetic properties of materials and media used to simulate EF and MF stimulations.

	Relative electric permittivity	Electric conductivity (S/m)	Relative magnetic permeability	Dimensions of domains
Electrodes	1	6.0 × 10^7^	1	Height (stripes): 2 × 12 mm, 2 × 20 mm, 2 × 25 mm, 2 × 28 mm, 2 × 30 mm, 2 × 31 mm; Width (stripes): 2 mm; Thickness (stripes): 1 mm; Radius (wire): 0.5 mm; Height (wire): 2 mm
Petri dishes	2.6	6.7 × 10^−14^	1	Radius: 17.5 mm; Height: 2 mm; Thickness (walls): 0.5 mm
Substrate	3	6.7 × 10^−14^	0.866	Radius: 17.5 mm; Height: 0.5 mm
Air	1	0	1	Radius: 25 mm; Height: 5 mm
Cellular medium (low cell confluence condition)	73	1.2 × 10^−7^	1	Radius: 17 mm; Height: 10 μm
Cellular medium (full cell confluence condition)	73	1.2 × 10^−7^	1	Radius: 17 mm; Height: 20 μm
Physiological medium	73	1.6	1	Radius: 17 mm; Height: 1 mm

## References

[b1] Chan K. . Musculoskeletal regeneration research network: A global initiative. J. Orthop. Trans. 3(4), 160–165 (2015). 10.1016/j.jot.2015.08.007 PMC5986990 30035054

[b2] March L. . Burden of disability due to musculoskeletal (MSK) disorders. Best Pract. Res. Cl. Rh. 28(3), 353–366 (2014). 10.1016/j.berh.2014.08.002 25481420

[b3] Pivec R., Johnson A. J., Mears S. C. & Mont M. A. Hip arthroplasty. Lancet 380, 1768–1777 (2012). 23021846 10.1016/S0140-6736(12)60607-2

[b4] Carr A. J. . Knee replacement. Lancet 379, 1331–1340 (2012). 22398175 10.1016/S0140-6736(11)60752-6

[b5] Kapadia B. H. . Periprosthetic joint infection. Lancet, 387, 386–394 (2016). 26135702 10.1016/S0140-6736(14)61798-0

[b6] Kurtz S., Ong K., Lau E., Mowat F. & Halpern M. Projections of primary and revision hip and knee arthroplasty in the United States from 2005 to 2030. J. Bone Joint Surg. 89(4), 780–785 (2007). 17403800 10.2106/JBJS.F.00222

[b7] Labek G., Thaler M., Janda W., Agreiter M. & Stöckl B. Revision rates after total joint replacement - cumulative results from worldwide joint register datasets. J. Bone Joint Surg. 93(3), 293–297 (2011). 10.1302/0301-620X.93B3.25467 21357948

[b8] Soares dos Santos M. P. . Instrumented hip joint replacements, femoral replacements and femoral fracture stabilizers. Expert Rev. Med. Devic. 11(6), 617–635 (2014). 10.1586/17434440.2014.946695 25234709

[b9] Torrão J., Soares dos Santos M. P. & Ferreira J. A. Instrumented knee joint implants: innovations and promising concepts. Expert Rev. Med. Devic. 12(5), 571–584 (2015). 10.1586/17434440.2015.1068114 26202322

[b10] Goriainov V., Cook R., Latham J. M., Dunlop D. G. & Oreffo R. O. Bone and metal: an orthopaedic perspective on osseointegration of metals. Acta Biomater. 10(10), 4043–4057 (2014). 24932769 10.1016/j.actbio.2014.06.004

[b11] Coelho P. G. & Jimbo R. Osseointegration of metallic devices: current trends based on implant hardware design. Arch. Biochem. Biophys. 561, 99–108 (2014). 25010447 10.1016/j.abb.2014.06.033

[b12] Coelho P. G. . Nanometer-scale features on micrometer-scale surface texturing: a bone histological, gene expression, and nanomechanical study. Bone 65, 25–32 (2014). 24813260 10.1016/j.bone.2014.05.004

[b13] Sumner D. R. Long-term implant fixation and stress-shielding in total hip replacement. J. Biomech. 48(5), 797–800 (2015). 25579990 10.1016/j.jbiomech.2014.12.021

[b14] Pakos E. E. . Long term outcomes of total hip arthroplasty with custom made femoral implants in patients with congenital disease of hip. J. Arthroplasty 30(12), 2242–2247 (2015). 26187384 10.1016/j.arth.2015.06.038

[b15] Bartolomé J. F. . *In vitro* and *in vivo* evaluation of a new zirconia/niobium biocermet for hard tissue replacement. Biomaterials 76, 313–320 (2016). 26561930 10.1016/j.biomaterials.2015.10.058

[b16] Soares dos Santos M. P., Ferreira J. A., Ramos A. & Simões J. A. Active orthopaedic implants: towards optimality. J. Frankl. Inst. 352(3), 813–834 (2015).

[b17] Goodman S. B., Yao Z., Keeney M. & Yang F. The future of biologic coatings for orthopaedic implants. Biomaterials 34(13), 3174–3183 (2013). 23391496 10.1016/j.biomaterials.2013.01.074 PMC3582840

[b18] Zhang B. G., Myers D. E., Wallace G. G., Brandt M. & Choong P. F. Bioactive coatings for orthopaedic implants - recent trends in development of implant coatings. Int. J. Mol. Sci. 15(7), 11878–11921 (2014). 25000263 10.3390/ijms150711878 PMC4139820

[b19] Goosen J. H., Kums A. J., Kollen B. J. & Verheyen C. C. Porous-coated femoral components with or without hydroxyapatite in primary uncemented total hip arthroplasty: a systematic review of randomized controlled trials. Arch. Orthop. Traum. Su. 129(9), 1165–1169 (2009). 10.1007/s00402-008-0749-9 18815799

[b20] Lyndon J. A., Boyd B. J. & Birbilis N. Metallic implant drug/device combinations for controlled drug release in orthopaedic applications. J. Controlled Release 179, 63–75 (2014). 10.1016/j.jconrel.2014.01.026 24512924

[b21] He J. . Collagen-infiltrated porous hydroxyapatite coating and its osteogenic properties: *In vitro* and *in vivo* study. J. Biomed. Mater. Res. A 100(7), 1706–1715 (2012). 22447757 10.1002/jbm.a.34121

[b22] Xu Q., Tanaka Y. & Czernuszka J. T. Encapsulation and release of a hydrophobic drug from hydroxyapatite coated liposomes. Biomaterials 28(16), 2687–2694 (2007). 17331574 10.1016/j.biomaterials.2007.02.007

[b23] Graichen F., Bergmann G. & Rohlmann A. Hip endoprosthesis for *in vivo* measurement of joint force and temperature. J. Biomech. 32(10), 1113–1117 (1999). 10476850 10.1016/s0021-9290(99)00110-4

[b24] Damm P., Graichen F., Rohlmann A., Bendera A. & Bergmann G. Total hip joint prosthesis for *in vivo* measurement of forces and moments. Med. Eng. Phys. 32(1), 95–100 (2010). 19889565 10.1016/j.medengphy.2009.10.003

[b25] Bergmann G. . High-tech hip implant for wireless temperature measurements *in vivo*. PLoS One 7(8), e43489 (2012). 22927973 10.1371/journal.pone.0043489 PMC3425470

[b26] Reis J. . A new piezoelectric actuator induces bone formation *in vivo*: a preliminary study. J. Biomed. Biotechnol. 2012, 1–7 (2012). 22701304 10.1155/2012/613403 PMC3369535

[b27] Aydin N. & Bezer M. The effect of an intramedullary implant with a static magnetic field on the healing of the osteotomised rabbit femur. Int. Orthop. 35(1), 135–141 (2011). 20062989 10.1007/s00264-009-0932-9 PMC3014488

[b28] Schmidt C., Zimmermann U. & van Rienen U. Modeling of an optimized electro- stimulative hip revision system under consideration of uncertainty in the conductivity of bone tissue. IEEE J. Biomed. Health Inform. 19(4), 1321–1330 (2015). 25898285 10.1109/JBHI.2015.2423705

[b29] Balint R., Cassidy N. J. & Cartmell S. H. Electrical stimulation: a novel tool for tissue engineering. Tissue Eng. Part B Rev. 19(1), 48–57 (2013). 22873689 10.1089/ten.TEB.2012.0183

[b30] Zhenyu W., Clark C. C. & Brighton C. T. Up-regulation of bone morphogenetic proteins in cultured murine bone cells with use of specific electric fields. J. Bone Joint Surg. Am. 88(5), 1053–1065 (2006). 16651580 10.2106/JBJS.E.00443

[b31] Brighton C. T., Wang W., Seldes R., Zhang G. & Pollack S. R. Signal transduction in electrically stimulated bone cells. J. Bone Joint Surg. Am. 83(10), 1514–1523 (2001). 11679602 10.2106/00004623-200110000-00009

[b32] Wiesmann H.-P., Hartig M., Stratmann U., Meyer U. & Joos U. Electrical stimulation influences mineral formation of osteoblast-like cells *in vitro*. BBA-Mol. Cell Res. 1538(1), 28–37 (2001). 10.1016/s0167-4889(00)00135-x 11341980

[b33] Hartig M., Joos U. & Wiesmann H. P. Capacitively coupled electric fields accelerate pro- liferation of osteoblast-like primary cells and increase bone extracellular matrix formation *in vitro*. Eur. Biophys. J. 29(7), 499–506 (2000). 11156291 10.1007/s002490000100

[b34] Griffin M., Iqbal S. A., Sebastian A., Colthurst J. & Bayat A. Degenerate wave and capacitive coupling increase human MSC invasion and proliferation while reducing cytotoxicity in an *in vitro* wound healing model. PLoS One 6(8), e23404 (2011). 21858102 10.1371/journal.pone.0023404 PMC3156742

[b35] Hronik-Tupaj M. & Kaplan D. L. A Review of the responses of two- and three-dimensional engineered tissues to electric fields. Tissue Eng. Part B Rev. 18(3), 167–180 (2012). 22046979 10.1089/ten.teb.2011.0244 PMC3357076

[b36] Vijayalaxmi Obe. G. Controversial cytogenetic observations in mammalian somatic cells exposed to extremely low frequency electromagnetic radiation: a review and future research recommendations. Bioelectromagnetics 26(5), 412–430 (2005). 15887256 10.1002/bem.20111

[b37] Lee M. S. . High-performance, transparent, and stretchable electrodes using graphene-metal nanowire hybrid structures. Nano Lett. 13(6), 2814–2821 (2013). 23701320 10.1021/nl401070p

[b38] Griffin M., Sebastian A., Colthurst J. & Bayat A. Enhancement of differentiation and mineralisation of osteoblast-like cells by degenerate electrical waveform in an *in vitro* electrical stimulation model compared to capacitive coupling. PLoS One 8(9), e72978 (2013). 24039834 10.1371/journal.pone.0072978 PMC3770651

[b39] Lavenus S. . Behaviour of mesenchymal stem cells, fibroblasts and osteoblasts on smooth surfaces. Acta Biomater. 7(4), 1525–1534 (2011). 21199693 10.1016/j.actbio.2010.12.033

[b40] Kang K. S., Hong. J. M., Kang J. A., Rhie J. W., Jeong Y. H. & Cho D. W. Regulation of osteogenic differentiation of human adipose-derived stem cells by controlling electromagnetic field conditions. Exp. Mol. Med. 45(e6), 1–9 (2013). 10.1038/emm.2013.11 PMC3584658 23306704

[b41] Soares dos Santos M. P. . Instrumented hip implants: Electric supply systems. J. Biomech. 46(15), 2561–2571 (2013). 24050511 10.1016/j.jbiomech.2013.08.002

[b42] Soares dos Santos M. P. . Magnetic levitation-based electromagnetic energy harvesting: a semi-analytical non-linear model for energy transduction. Sci. Rep. 6, 18579 (2016). 26725842 10.1038/srep18579 PMC4698582

[b43] Silva N. M. . Power management architecture for smart hip prostheses comprising multiple energy harvesting systems. Sensor. Actuat. A-Phys. 202(1), 183–192 (2013).

[b44] Ozawa H., Abe E., Shibasaki Y., Fukuhara T. & Suda T. Electric fields stimulate DNA synthesis of mouse osteoblast-like cells (MC3T3-El) by a mechanism involving calcium ions. J. Cell. Physiol. 138(3), 477–483 (1989). 2538484 10.1002/jcp.1041380306

[b45] Tomaselli V. P. & Shamos M. H. Electrical properties of hydrated collagen. I. dielectric properties. Biopolymers 12(2), 353–366 (1973). 10.1002/bip.1974.360131203 4471612

[b46] Tomaselli V. P. & Shamos M. H. Electrical Properties of Hydrated Collagen. II. semi- conductor properties. Biopolymers 13(12), 2423–2434 (1974). 4471612 10.1002/bip.1974.360131203

[b47] Pucihar G., Kotnik T., Kanduser M. & Miklavčič D. The influence of medium conductivity on electropermeabilization and survival of cells *in vitro*. Bioelectrochemistry 54(2), 107–115 (2001). 11694390 10.1016/s1567-5394(01)00117-7

[b48] Quarles L. D., Yohay D. A., Lever L. W., Caton R. & Wenstrup R. J. Distinct proliferative and differentiated stages of murine MC3T3-E1 cells in culture: an *in vitro* model of osteoblast development. J. Bone Miner. Res. 7(6), 683–92 (1992). 1414487 10.1002/jbmr.5650070613

[b49] Zhuang H. . Electrical stimulation induces the level of TGF-β1 mRNA in osteoblastic cells by a mechanism involving calcium/calmodulin pathway. Biochem. Biophys. Res. Commun. 237(2), 225–229 (1997). 9268690 10.1006/bbrc.1997.7118

[b50] Fitzsimmons R. J., Strong D. D., Mohan S. & Baylink D. J. Low-amplitude, low- frequency electric field-stimulated bone cell proliferation may in part be mediated by increased IGF-II release. J. Cell Physiol. 150(1), 84–89 (1992). 1730789 10.1002/jcp.1041500112

[b51] Fitzsimmons R. J., Farley J. R., Adey W. R. & Baylink D. J. Frequency dependence of increased cell proliferation, *in vitro*, in exposures to a low-amplitude, low-frequency electric field: evidence for dependence on increased mitogen activity released into culture medium. J. Cell. Physiol. 139(3), 586–591 (1989). 2738103 10.1002/jcp.1041390319

[b52] Creecy C. M. . Mesenchymal stem cell osteodifferentiation in response to alternating electric current. Tissue Eng. Part A. 19(3–4), 467–474 (2013). 23083071 10.1089/ten.tea.2012.0091 PMC3542886

[b53] Hronik-Tupaj M., Rice W. L., Cronin-Golomb M., Kaplan D. L. & Georgakoudi I. Osteoblastic differentiation and stress response of human mesenchymal stem cells exposed to alternating current electric fields. Biomed. Eng. Online 10(9), 1–22 (2011). 21269490 10.1186/1475-925X-10-9 PMC3039627

[b54] Selvamurugan N., Kwok S., Vasilov A., Jefcoat S. C. & Partridge N. C. Effects of BMP-2 and pulsed electromagnetic field (PEMF) on rat primary osteoblastic cell proliferation and gene expression. J. Orthop. Res. 25(9), 1213–1220 (2007). 17503520 10.1002/jor.20409

[b55] Zhou J. . Effects of 50 Hz sinusoidal electromagnetic fields of different intensities on proliferation, differentiation and mineralization potentials of rat osteoblasts. Bone 49(4), 753–761 (2011). 21726678 10.1016/j.bone.2011.06.026

[b56] Pina A. . *In Vitro* performance assessment of new brushite-forming Zn- and ZnSr- substituted β-TCP bone cements. J. Biomed. Mater. Res. B Appl. Biomater. 94(2), 414–420 (2010). 20574977 10.1002/jbm.b.31669

[b57] Henriques A. G., Vieira S. I., da Cruz e Silva E. F. & Cruz e Silva O. A. Aβ hinders nuclear targeting of AICD and Fe65 in primary neuronal cultures. J. Mol. Neurosci. 39 (1–2), 248–255 (2009). 19340611 10.1007/s12031-009-9192-9 PMC2744832

[b58] Rocha J. F., da Cruz e Silva O. A. & Vieira S. I. Analysis of the Amyloid Precursor Protein (APP) role in neuritogenesis reveals a biphasic SH-SY5Y neuronal cell differentiation model. J. Neurochem. 134(3), 288–301 (2015). 25903790 10.1111/jnc.13133

[b59] Torres P. M. . Effects of Mn-doping on the structure and biological properties of β-tricalcium phosphate. J. Inorg. Biochem. 136, 57–66 (2014). 24747361 10.1016/j.jinorgbio.2014.03.013

[b60] Gregory C. A., Gunn W. G., Peister A. & Prockop D. J. An Alizarin red-based assay of mineralization by adherent cells in culture: comparison with cetylpyridinium chloride extraction. Anal. Biochem. 329(1), 77–84 (2004). 15136169 10.1016/j.ab.2004.02.002

