# Supplementary material for: New cosurface capacitive stimulators for the development of active osseointegrative implantable devices
Source: Sci Rep. 2016 Jul 26;6:30231. doi: 10.1038/srep30231 (PMC4960616; doi:10.1038/srep30231)
Supplement: Supplementary Information [file srep30231-s1.pdf]

New cosurface capacitive stimulators for the development of active osseointegrative implantable devices

Supplementary material

Marco P. Soares dos Santos[1,2], Ana Marote[3], T. Santos[4], João Torrão[1,2], A. Ramos[1,2], José A. O. Simões[2], Odete A. B. da Cruz e Silva[3], Edward P. Furlani[5,6], Sandra I. Vieira[3] and Jorge A. F. Ferreira[1,2]

[1] Centre for Mechanical Technology and Automation (TEMA), Universidade de Aveiro, Aveiro, Portugal.
[2] Department of Mechanical Engineering, Universidade de Aveiro, Aveiro, Portugal.
[3] Institute for Biomedicine (iBiMED), Department of Medical Sciences, Universidade de Aveiro, Aveiro, Portugal
[4] Physics Department, University of Aveiro, Aveiro, Portugal
[5] Department of Chemical and Biological Engineering, University at Buffalo, SUNY, Buffalo, NY, US
[6] Department of Electrical Engineering, University at Buffalo, SUNY, Buffalo, NY, US

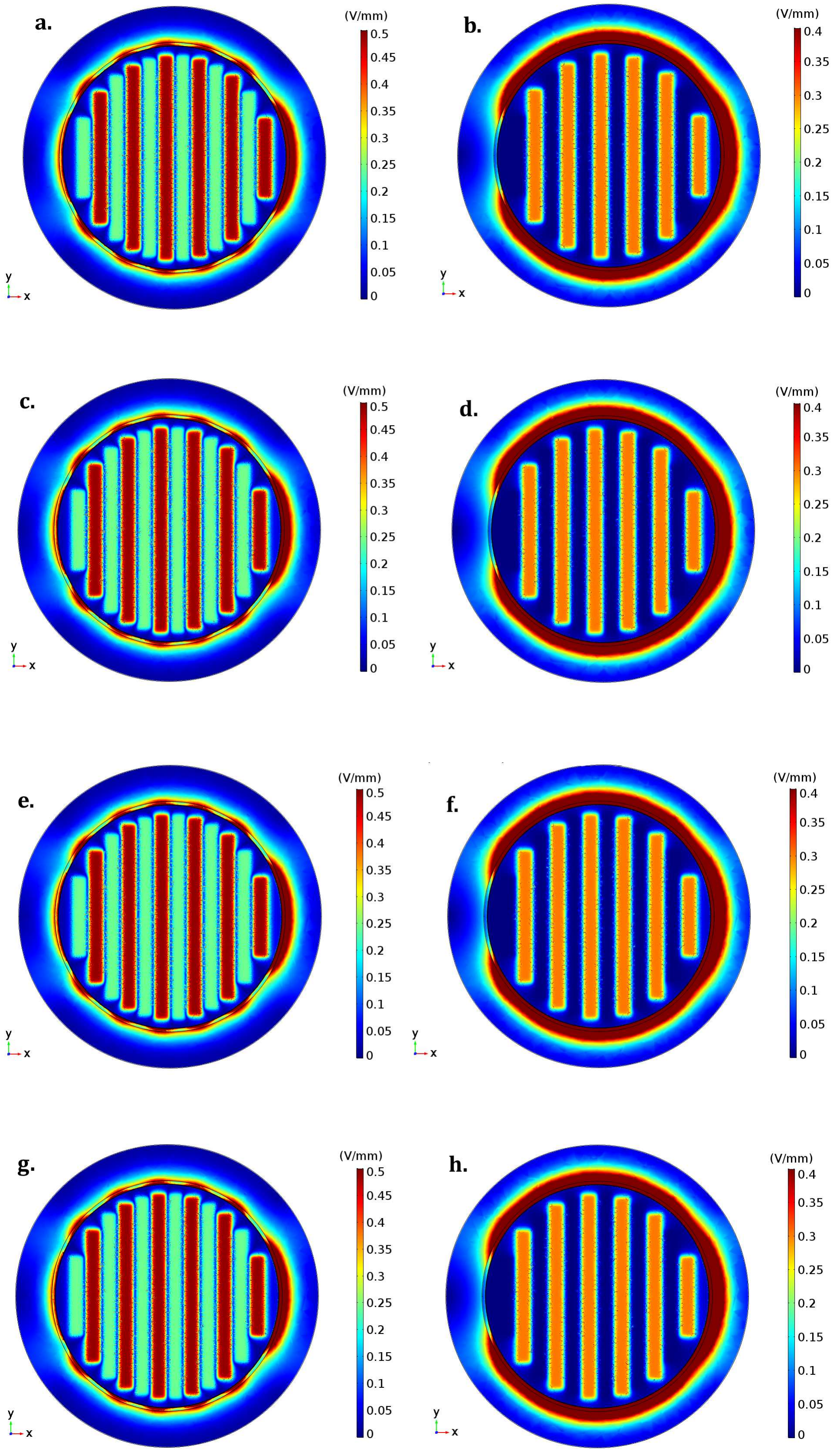

Fig. S1: Simulation results of EF distributions and strengths: **a**. 2D EFs stimulating cells at π rad: HF ST, low cell confluence condition, z=0.5005 mm; **b**. 2D EFs stimulating cells at π rad: LF ST, low cell confluence condition, z=0.5005 mm; **c**. 2D EFs stimulating the cellular tissue at π rad: HF ST, full cell confluence condition, z=0.5005 mm; **d**. 2D EFs stimulating the cellular tissue at π rad: LF ST, full cell confluence condition, z=0.5005 mm; **e**. 2D EFs stimulating cells at π rad: HF ST, low cell confluence condition, z=0.5095 mm; **f**. 2D EFs stimulating cells at π rad: LF ST, low cell confluence condition, z=0.5095 mm; **g**. 2D EFs stimulating the cellular tissue at π rad: HF ST, full cell confluence condition, z=0.5195 mm; **h**. 2D EFs stimulating the cellular tissue at π rad: LF ST, full cell confluence condition, z=0.5195 mm.

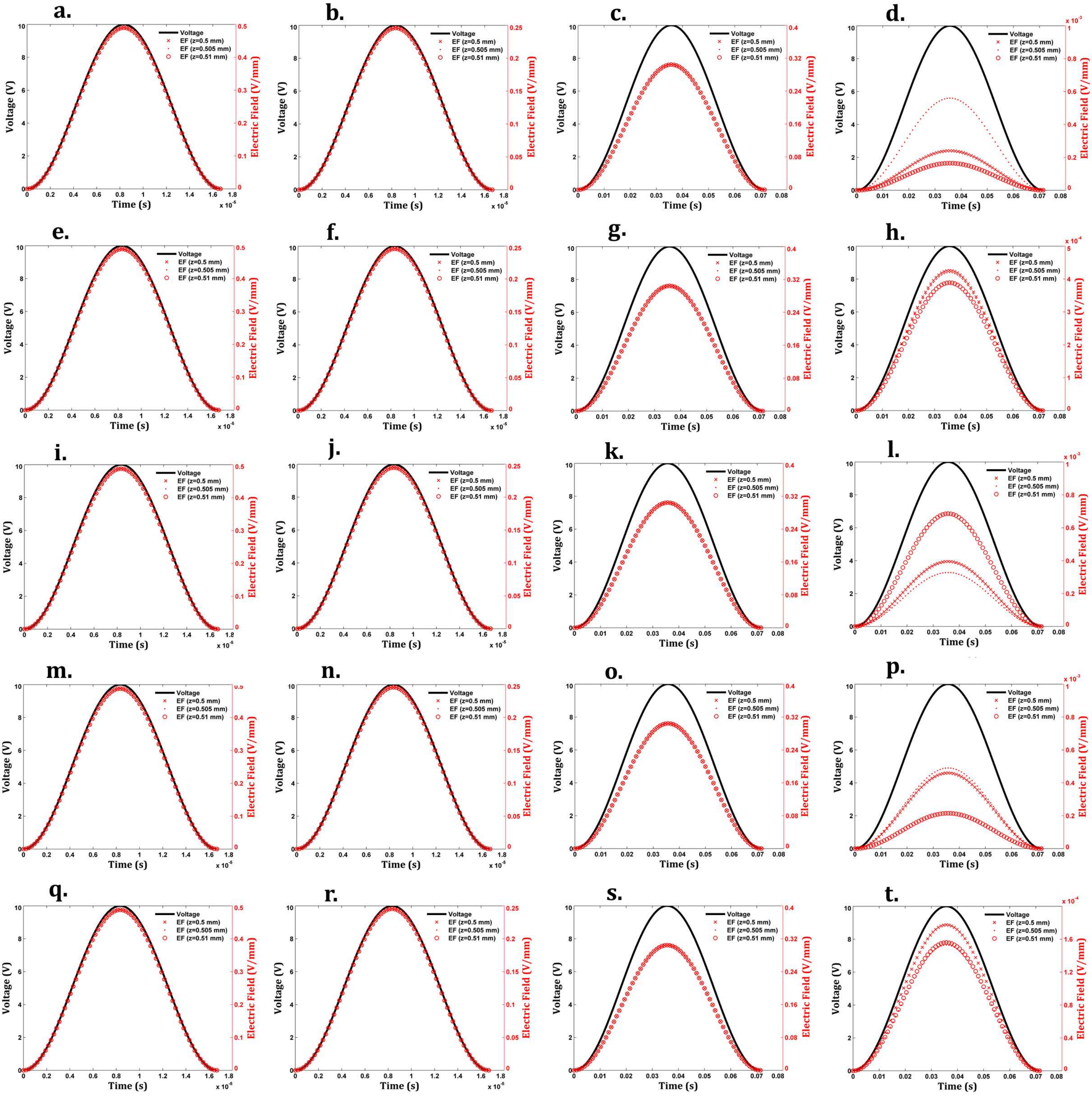
a.
b.
c.
d.
e.
f.
g.
h.
i.
j.
k.
l.
m.
n.
o.
p.
q.
r.
s.
t.
Voltage
EF (z=0.5 mm)
EF (z=0.505 mm)
EF (z=0.51 mm)
Voltage (V)
Electric Field (V/mm)
Time (s)

Fig S2: Simulation results of EF dynamics. a. EFs in [0 2π] rad: HF ST at (x,y,x)=(-3.75,0,0.5005) mm, (x,y,x)=(-3.75,0,0.505) mm and (x,y,x)=(-3.75,0,0.5095) mm; **b.** EFs in [0 2π] rad: HF ST at (x,y,x)=(3.75,0,0.5005) mm, (x,y,x)=(3.75,0,0.505) mm and (x,y,x)=(3.75,0,0.5095) mm; **c.** EFs in [0 2π] rad: LF ST at (x,y,x)=(-3.75,0,0.5005) mm, (x,y,x)=(-3.75,0,0.505) mm and (x,y,x)=(-3.75,0,0.5095) mm; **d.** EFs in [0 2π] rad: LF ST at (x,y,x)=(3.75,0,0.5005) mm, (x,y,x)=(3.75,0,0.505) mm and (x,y,x)=(3.75,0,0.5095) mm; **e.** EFs in [0 2π] rad: HF ST at (x,y,x)=(-6.25,0,0.5005) mm, (x,y,x)=(-6.25,0,0.505) mm and (x,y,x)=(-6.25,0,0.5095) mm; **f.** EFs in [0 2π] rad: HF ST at (x,y,x)=(6.25,0,0.5005) mm, (x,y,x)=(6.25,0,0.505) mm and (x,y,x)=(6.25,0,0.5095) mm; **g.** EFs in [0 2π] rad: LF ST at (x,y,x)=(-6.25,0,0.5005) mm, (x,y,x)=(-6.25,0,0.505) mm and (x,y,x)=(-6.25,0,0.5095) mm; **h.** EFs in [0 2π] rad: LF ST at (x,y,x)=(6.25,0,0.5005) mm, (x,y,x)=(6.25,0,0.505) mm and (x,y,x)=(6.25,0,0.5095) mm; **i.** EFs in [0 2π] rad: HF ST at (x,y,x)=(-8.75,0,0.5005) mm, (x,y,x)=(-8.75,0,0.505) mm and (x,y,x)=(-8.75,0,0.5095) mm; **j** EFs in [0 2π] rad: HF ST at (x,y,x)=(8.75,0,0.5005) mm, (x,y,x)=(8.75,0,0.505) mm and (x,y,x)=(8.75,0,0.5095) mm; **k.** EFs in [0 2π] rad: LF ST at (x,y,x)=(-8.75,0,0.5005) mm, (x,y,x)=(-8.75,0,0.505) mm and (x,y,x)=(-8.75,0,0.5095) mm; **l.** EFs in [0 2π] rad: LF ST at (x,y,x)=(8.75,0,0.5005) mm, (x,y,x)=(8.75,0,0.505) mm and (x,y,x)=(8.75,0,0.5095) mm; **m.** EFs in [0 2π] rad: HF ST at (x,y,x)=(-11.25,0,0.5005) mm, (x,y,x)=(-11.25,0,0.505) mm and (x,y,x)=(-11.25,0,0.5095) mm; **n** EFs in [0 2π] rad: HF ST at (x,y,x)=(11.25,0,0.5005) mm, (x,y,x)=(11.25,0,0.505) mm and (x,y,x)=(11.25,0,0.5095) mm; **o.** EFs in [0 2π] rad: LF ST at (x,y,x)=(-11.25,0,0.5005) mm, (x,y,x)=(-11.25,0,0.505) mm and (x,y,x)=(-11.25,0,0.5095) mm; **p.** EFs in [0 2π] rad: LF ST at (x,y,x)=(11.25,0,0.5005) mm, (x,y,x)=(11.25,0,0.505) mm and (x,y,x)=(11.25,0,0.5095) mm; **q.** EFs in [0 2π] rad: HF ST at (x,y,x)=(-13.75,0,0.5005) mm, (x,y,x)=(-13.75,0,0.505) mm and (x,y,x)=(-13.75,0,0.5095) mm; **r** EFs in [0 2π] rad: HF ST at (x,y,x)=(13.75,0,0.5005) mm, (x,y,x)=(13.75,0,0.505) mm and (x,y,x)=(13.75,0,0.5095) mm; **s.** EFs in [0 2π] rad: LF ST at (x,y,x)=(-13.75,0,0.5005) mm, (x,y,x)=(-13.75,0,0.505) mm and (x,y,x)=(-13.75,0,0.5095) mm; **t.** EFs in [0 2π] rad: LF ST at (x,y,x)=(13.75,0,0.5005) mm, (x,y,x)=(13.75,0,0.505) mm and (x,y,x)=(13.75,0,0.5095) mm. All these results were obtained for the low cell confluence condition.

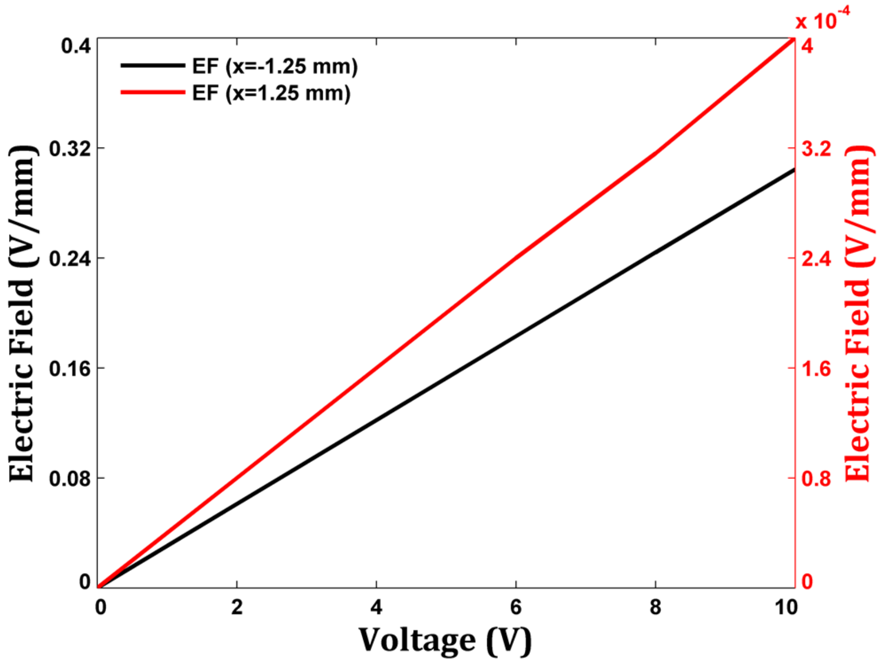

Fig S3: Approximate linear interrelationship between peak-to-peak LF EX (at π rad) and peak-to-peak EF strengths at (x,y,z)=(-1.25, 0, 0.505) mm and (x,y,z)=(1.25, 0, 0.505) mm. These results were obtained for the low cell confluence condition.

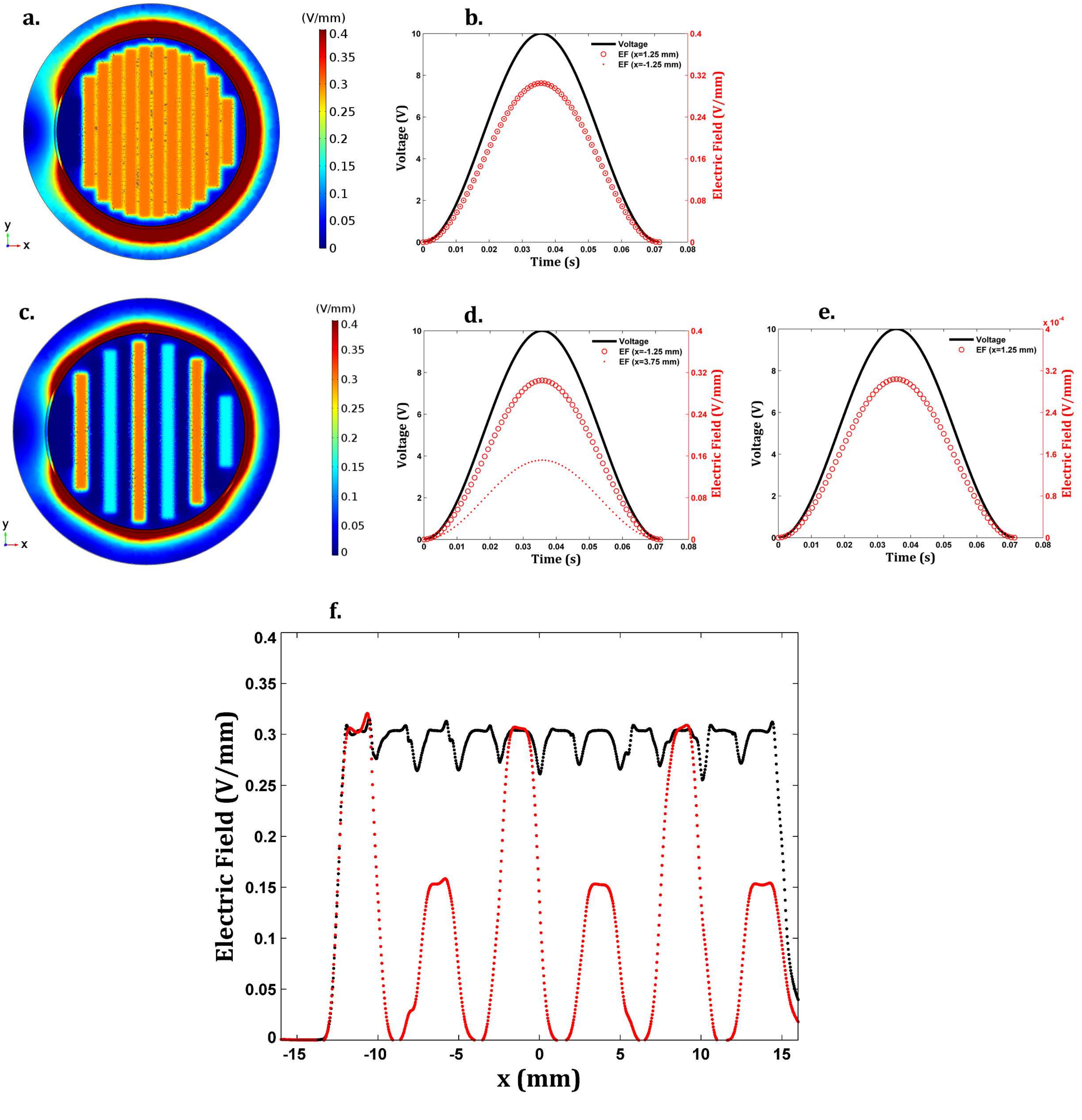

Fig S4: Simulation results of EF strengths and dynamics when different excitations are applied to the electrodes. **a.** and **b.** $K_{EX} = 5$ for all electrodes, apart from the one at the left extremity, for which $K_{EX} = 0$ was applied. **a.** 2D EFs stimulating cells at π rad: LF ST, z=0.505 mm; **b.** EFs in $[0\ 2\pi]$ rad: LF ST at (x,y,x)=(-1.25, 0, 0.505) mm and (x,y,x)=(1.25, 0, 0.505) mm; **c.** to **e.** $K_{EX} = 0$, $K_{EX} = 5$, $K_{EX} = 0$, $K_{EX} = 2.5$, $K_{EX} = 0$, $K_{EX} = 5$, $K_{EX} = 0$, $K_{EX} = 2.5$, $K_{EX} = 0$, $K_{EX} = 5$, $K_{EX} = 0$ and $K_{EX} = 2.5$ were applied to the electrodes, respectively from the one at the left extremity to one at the right extremity. **c.** 2D EFs stimulating cells at π rad: LF ST, z=0.505 mm; **d.** EFs in $[0\ 2\pi]$ rad: LF ST at (x,y,x)=(-1.25, 0, 0.505) mm and (x,y,x)=(3.75, 0, 0.505) mm; **e.** EFs in $[0\ 2\pi]$ rad: LF ST at (x,y,x)=(1.25, 0, 0.505) mm. **f.** EFs stimulating cells at π rad along the x-axis: (black) results from **a.** and **b.**; (red) results from **c.** to **e.** All these results were obtained for the low cell confluence condition.

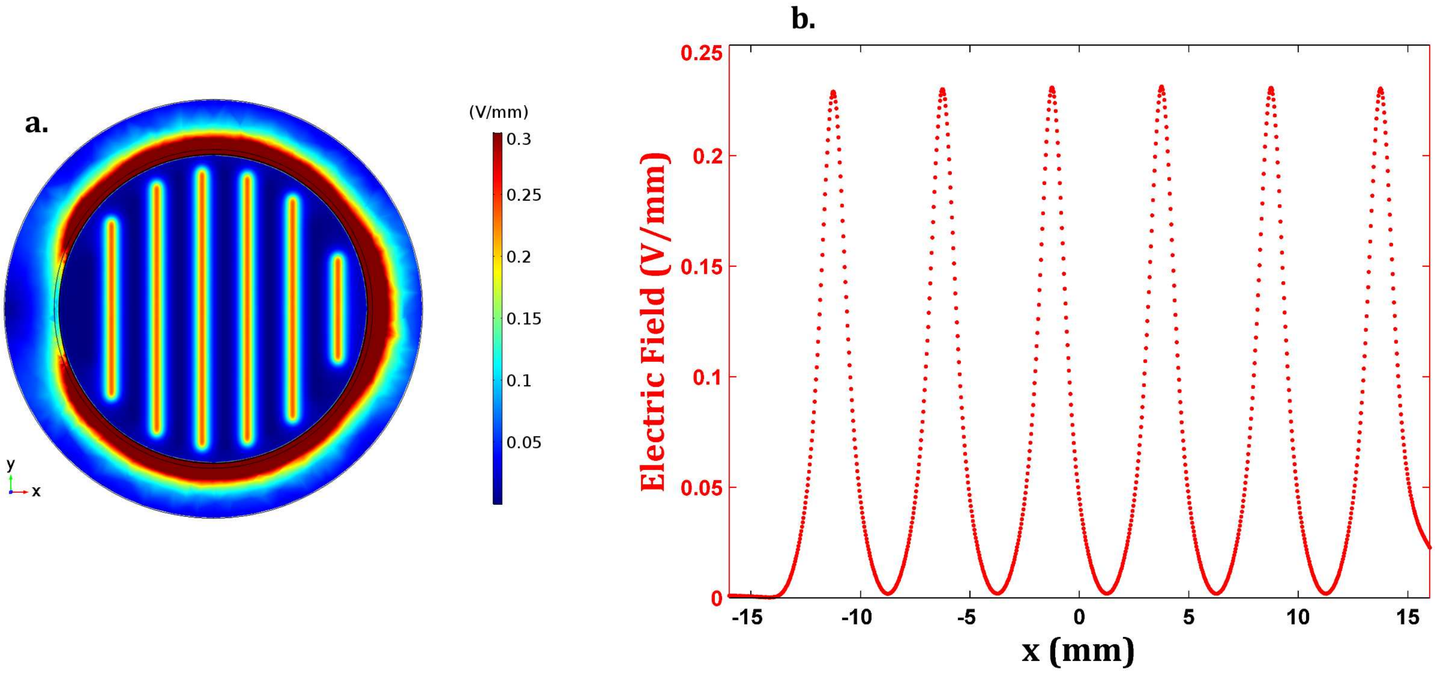

Fig S5: Simulation results of EFs stimulating cells by employing fillets with 0.45 mm radius to all corners of electrodes that are 1 mm wide. a. 2D EFs stimulating cells at π rad: LF ST, z=0.505 mm; **b.** EFs stimulating cells at π rad along the x-axis: LF ST, z=0.505 mm. These results were obtained for the low cell confluence condition.

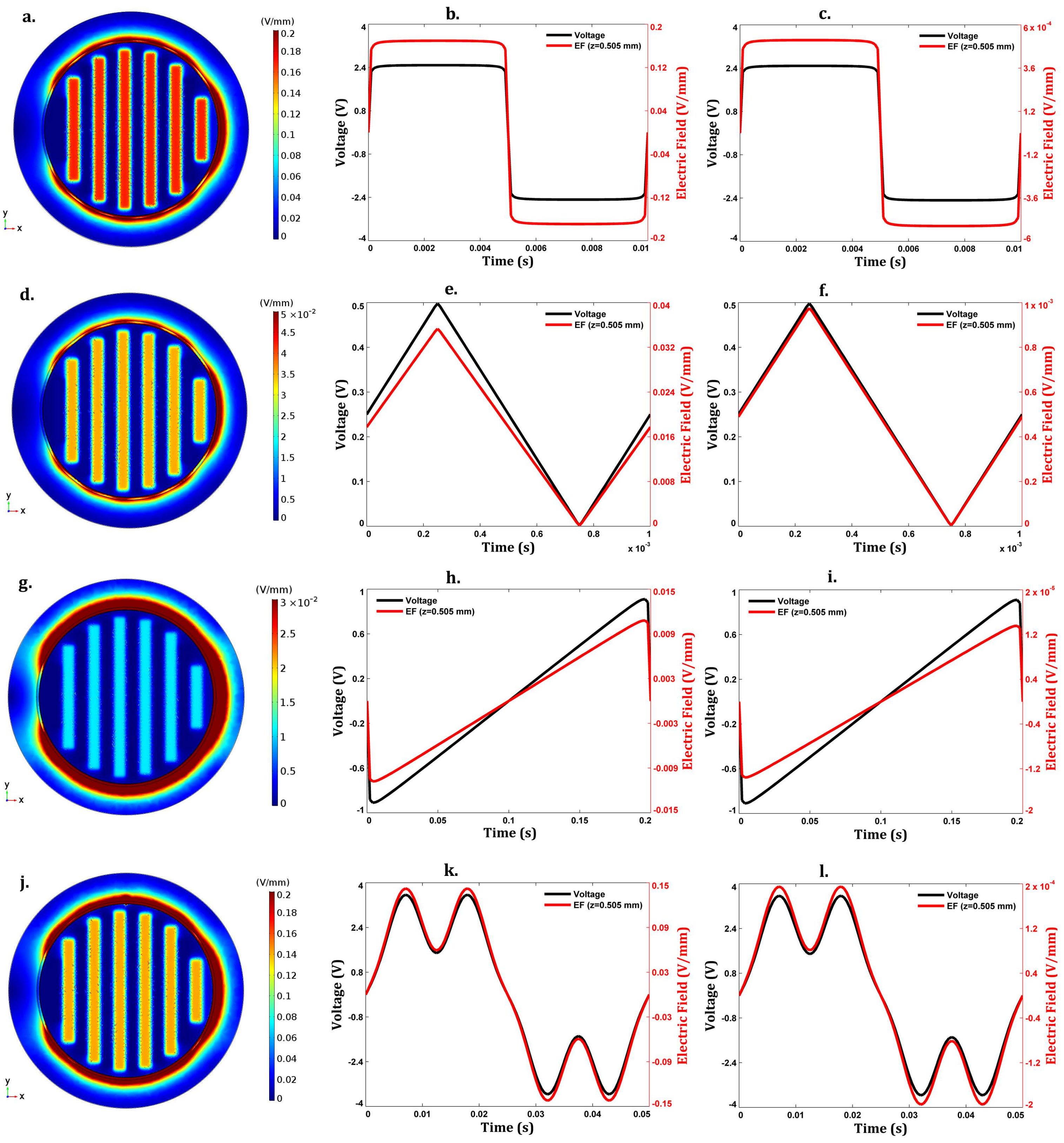

Fig. S6: Simulation results of EF strengths and dynamics, according to the following periodic excitations applied to stimulator' electrodes: a. 2D EFs stimulating cells at π rad: squared excitation (SQ ST) (5 V, 100 Hz), z=0.505 mm; **b.** EFs in [0 2π] rad: SQ ST at (x,y,x)=(-1.25,0,0.505) mm; **c.** EFs in [0 2π] rad: SQ ST at (x,y,x)=(1.25,0,0.505) mm; **d.** 2D EFs stimulating cells at π rad: triangular excitation (TR ST) (0.5 V, 1 Hz), z=0.505 mm; **e.** EFs in [0 2π] rad: TR ST at (x,y,x)=(-1.25,0,0.505) mm; **f.** EFs in [0 2π] rad: TR ST at (x,y,x)=(1.25,0,0.505) mm; **g.** 2D EFs stimulating cells at π rad: sawtooth excitation (SW ST) (2 V, 5 Hz), z=0.505 mm; **h.** EFs in [0 2π] rad: SW ST at (x,y,x)=(-1.25,0,0.505) mm; **i.** EFs in [0 2π] rad: SW ST at (x,y,x)=(1.25,0,0.505) mm; **j.** 2D EFs stimulating cells at π rad: arbitrary excitation (AR ST) (3.5 V, 20 Hz), z=0.505 mm; **k.** EFs in [0 2π] rad: AR ST at (x,y,x)=(-1.25,0,0.505) mm; **l.** EFs in [0 2π] rad: AR ST at (x,y,x)=(1.25,0,0.505) mm. Cross-correlations near 100% between all the excitation waveforms and all the generated EF waveforms were observed. These results were obtained for the low cell confluence condition.

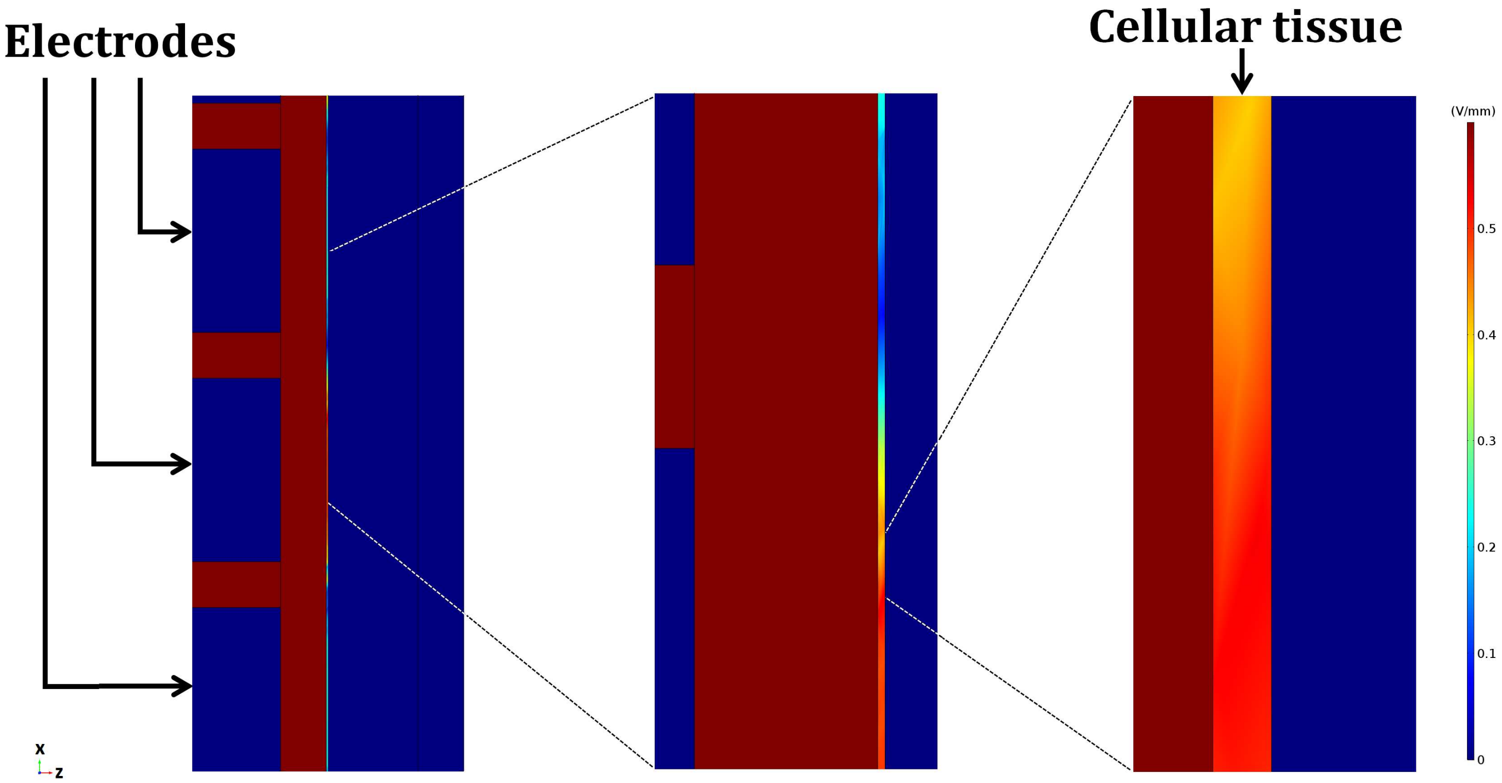

Fig. S7: Simulation results of EF strengths in the *xz*-plane. These results were obtained for the full cell confluence condition, HF ST, y=0 mm. Similar results were found in the *yz*-plane (x=0 mm).

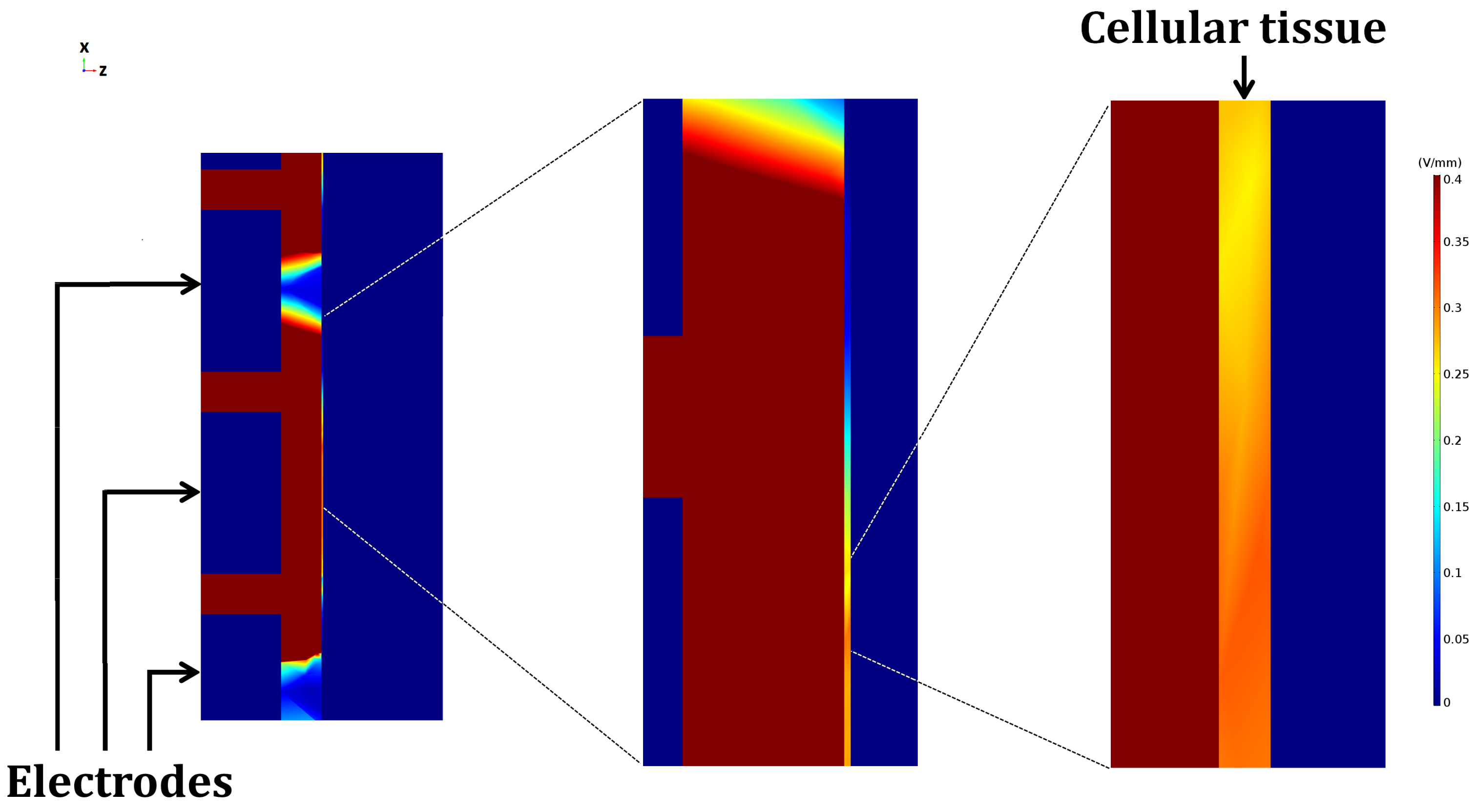

Fig. S8: Simulation results of EF strengths in the *xz*-plane. These results were obtained for the full cell confluence condition, LF ST, y=0 mm. Similar results were found in the *yz*-plane (x=0 mm).

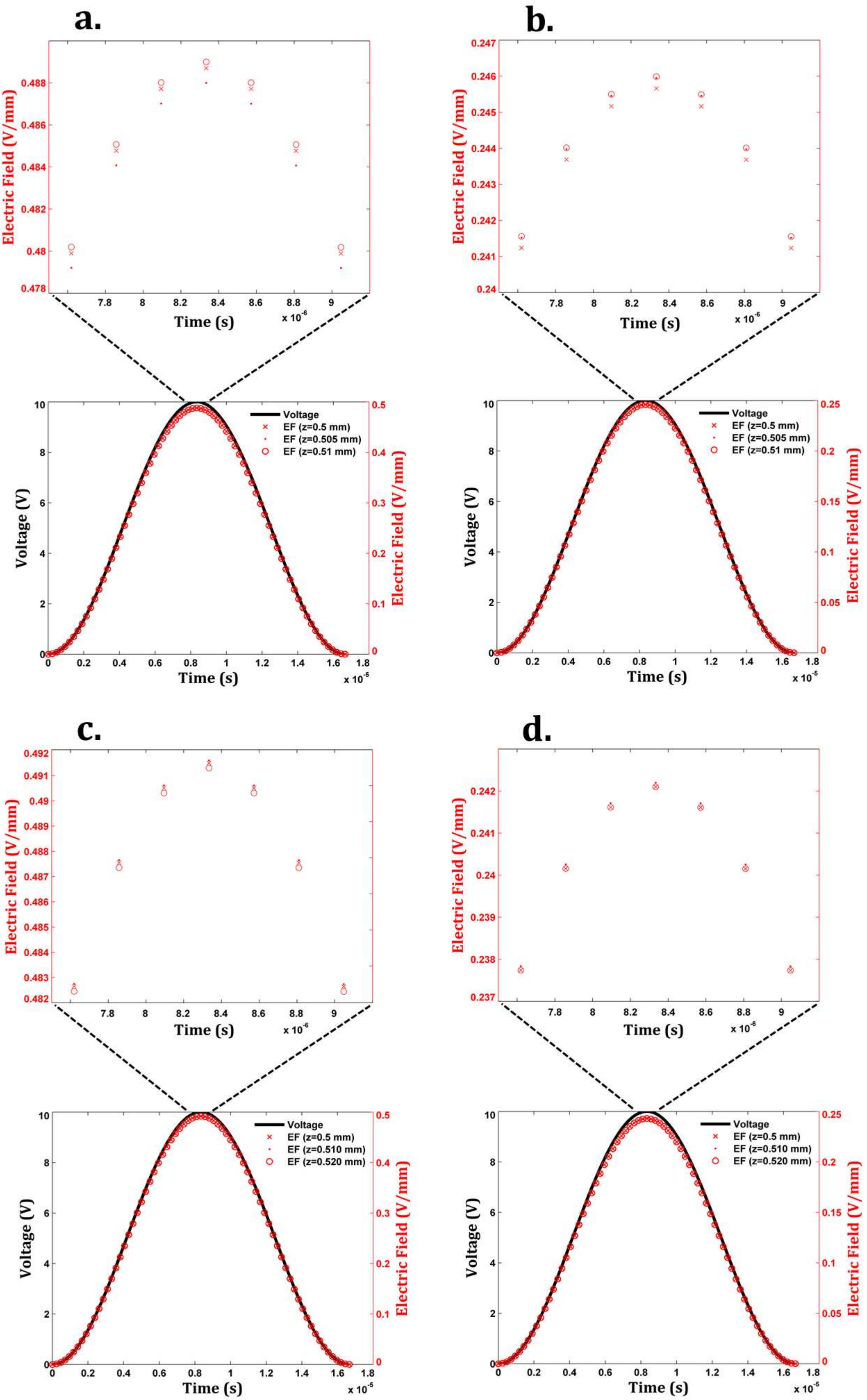

a.
Electric Field (V/mm)
Time (s)
x 10^-6
Voltage
EF (z=0.5 mm)
EF (z=0.505 mm)
EF (z=0.51 mm)
Voltage (V)
Electric Field (V/mm)
Time (s)
x 10^-5
b.
Electric Field (V/mm)
Time (s)
x 10^-6
Voltage
EF (z=0.5 mm)
EF (z=0.505 mm)
EF (z=0.51 mm)
Voltage (V)
Electric Field (V/mm)
Time (s)
x 10^-5
c.
Electric Field (V/mm)
Time (s)
x 10^-6
Voltage
EF (z=0.5 mm)
EF (z=0.510 mm)
EF (z=0.520 mm)
Voltage (V)
Electric Field (V/mm)
Time (s)
x 10^-5
d.
Electric Field (V/mm)
Time (s)
x 10^-6
Voltage
EF (z=0.5 mm)
EF (z=0.510 mm)
EF (z=0.520 mm)
Voltage (V)
Electric Field (V/mm)
Time (s)
x 10^-5

Fig. S9: Detailed analyses to the EF strengths stimulating cells and cellular tissue along the *z*-axis. a. EFs in [0 2π] rad: HF ST at (x,y,x)=(-1.25,0,0.5005) mm, (x,y,x)=(-1.25,0,0.505) mm and (x,y,x)=(-1.25,0,0.5095) mm, low cell confluence condition; **b**. EFs in [0 2π] rad: HF ST at (x,y,x)=(1.25,0,0.5005) mm, (x,y,x)=(1.25,0,0.505) mm and (x,y,x)=(1.25,0,0.5095) mm, low cell confluence condition; **c**. EFs in [0 2π] rad: HF ST at (x,y,x)=(-1.25,0,0.5005) mm, (x,y,x)=(-1.25,0,0.51) mm and (x,y,x)=(-1.25,0,0.5195) mm, full cell confluence condition; **d**. EFs in [0 2π] rad: HF ST at (x,y,x)=(1.25,0,0.5005) mm, (x,y,x)=(1.25,0,0.51) mm and (x,y,x)=(1.25,0,0.5195) mm, full cell confluence condition.

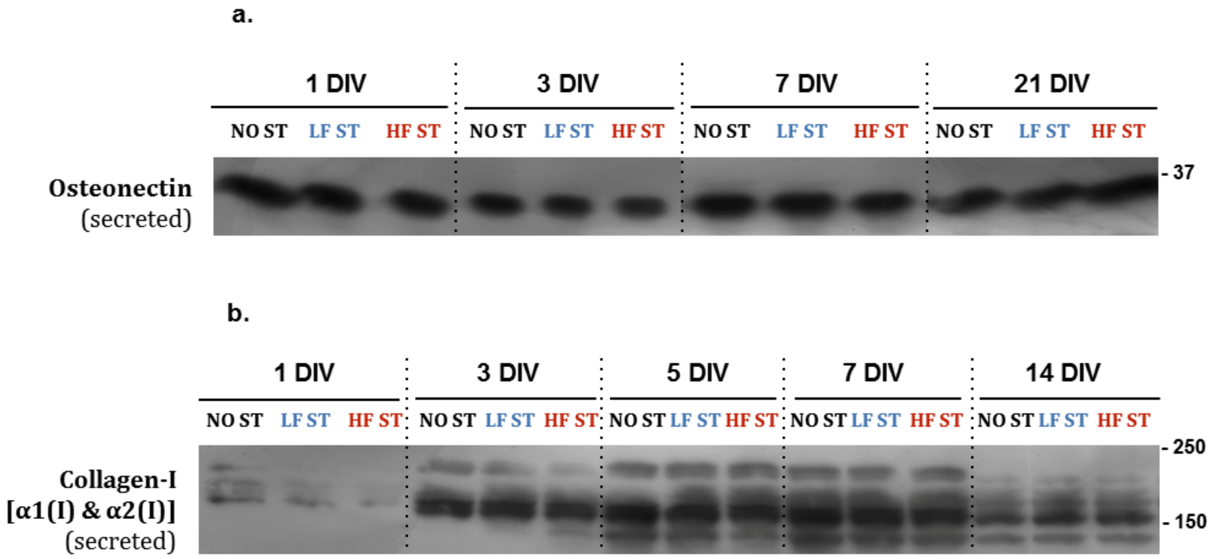

Fig. S10: Secretion of two matrix maturation markers into the cells conditioned media upon LF and HF stimuli Immunoblot analysis of the levels of: **a.** osteonectin (a 35 kDa protein); and **b.** collagen-I (unprocessed and processed $\alpha 1(\mathrm{I})$ and $\alpha 2(\mathrm{I})$ procollagen monomeric chains of 130-160 kDa)), secreted into the medium by MC3T3 cells exposed for various DIV to NO ST, LF ST and HF ST. Migration of molecular weight markers is indicated to the right.
